# Supplementary material for: The pace of shifting seasons in lakes
Source: Nat Commun. 2023 Apr 13;14:2101. doi: 10.1038/s41467-023-37810-4 (PMC10102225; doi:10.1038/s41467-023-37810-4)
Supplement: Supplementary file 1 — Supplementary Information [file 41467_2023_37810_MOESM1_ESM.docx]

**Supplementary Information**

**The pace of shifting seasons in lakes**

**Woolway**

**
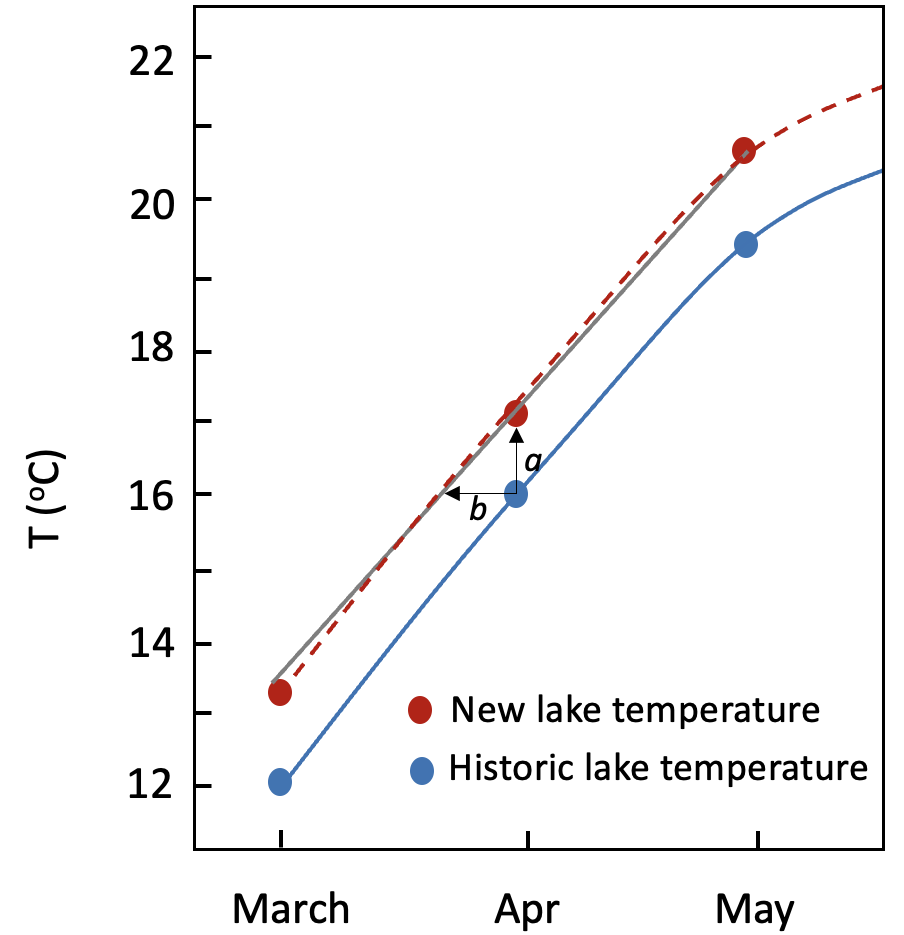
**

**Figure S1 |** Methodology for calculating the pace of shifting seasons in lakes. A seasonal shift is defined as the advance or delay in the seasonal timing of fixed temperatures. The seasonal shift in temperature (shown as *b*) is thus calculated by dividing the temperature change (shown as *a*) by the slope of temperature change. Pace of seasonal shift: *b* = *a*/slope. The example shown applies for spring.

**
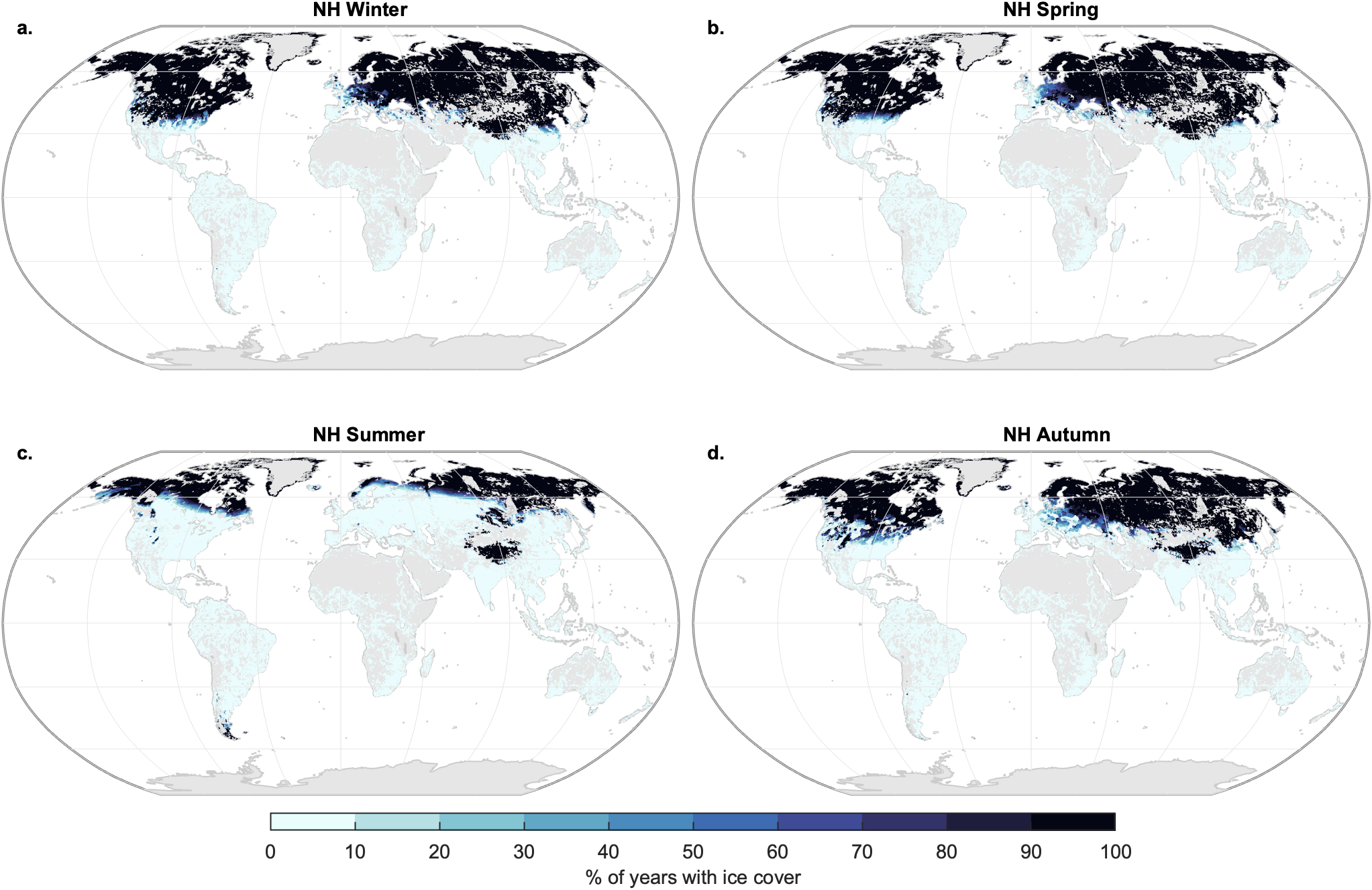
**

**Figure S2 |** Shown is the percentage of years (1980-2021) where lakes worldwide experience ice cover during (**a**) Winter (December-February in the Northern Hemisphere [NH] and June-August in Southern Hemisphere [SH]), (**b**) Spring (March-May in NH and September-November in the SH), (**c**) Summer (June-August in the NH and December-February in SH), and (**d**) Autumn (September-November in the NH and March-May in SH).

**
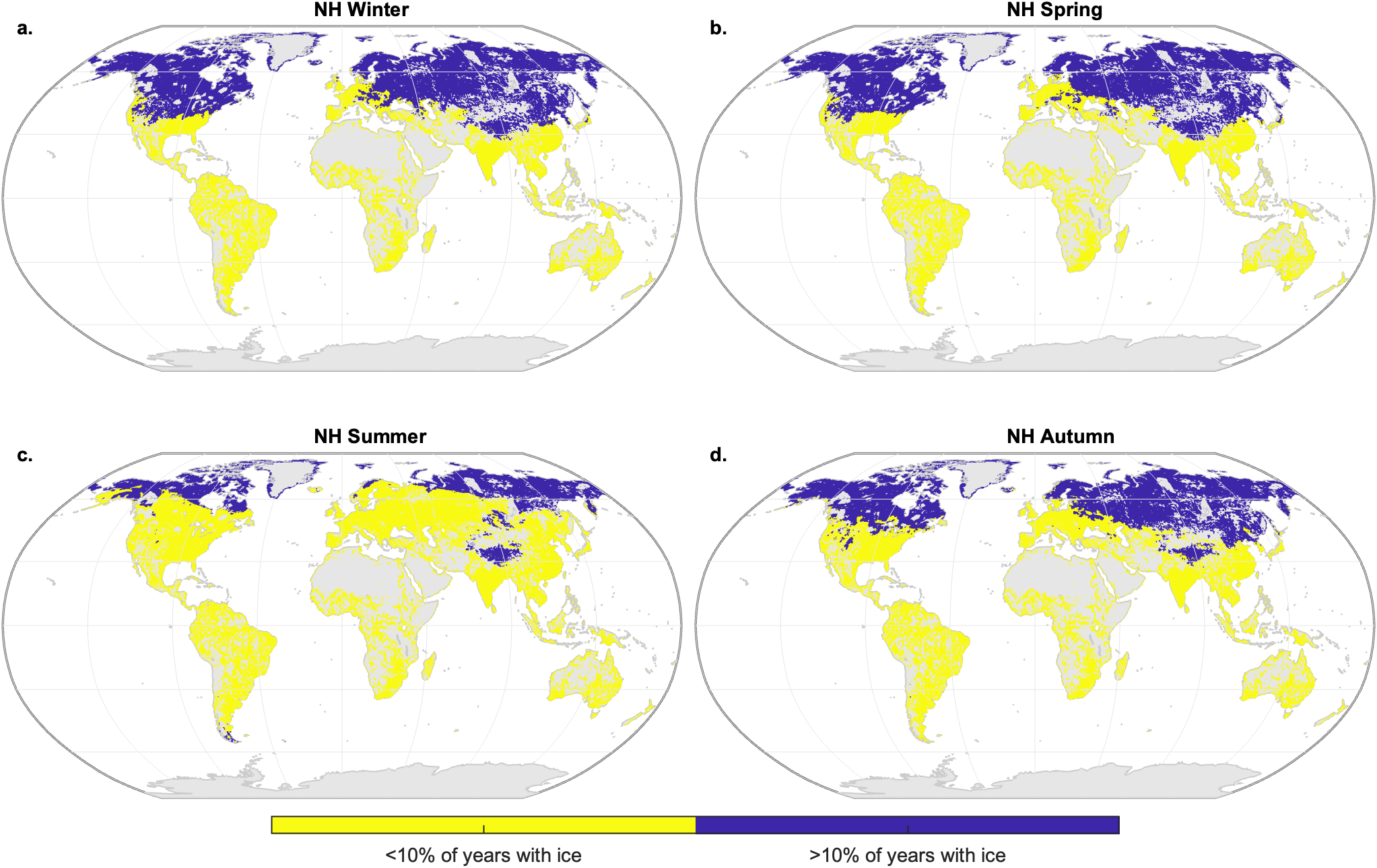
**

**Figure S3 |** Shown are lake regions that experience ice cover for more (blue) or less (yellow) than 10% of all years during the historic to contemporary period (1980-2021) for (**a**) Winter (December-February in the Northern Hemisphere [NH] and June-August in the Southern Hemisphere [SH]), (**b**) Spring (March-May in NH and September-November in the SH), (**c**) Summer (June-August in the NH and December-February in SH), and (**d**) Autumn (September-November in the NH and March-May in SH). Lakes in blue are excluded from the investigation during the season of interest.

**
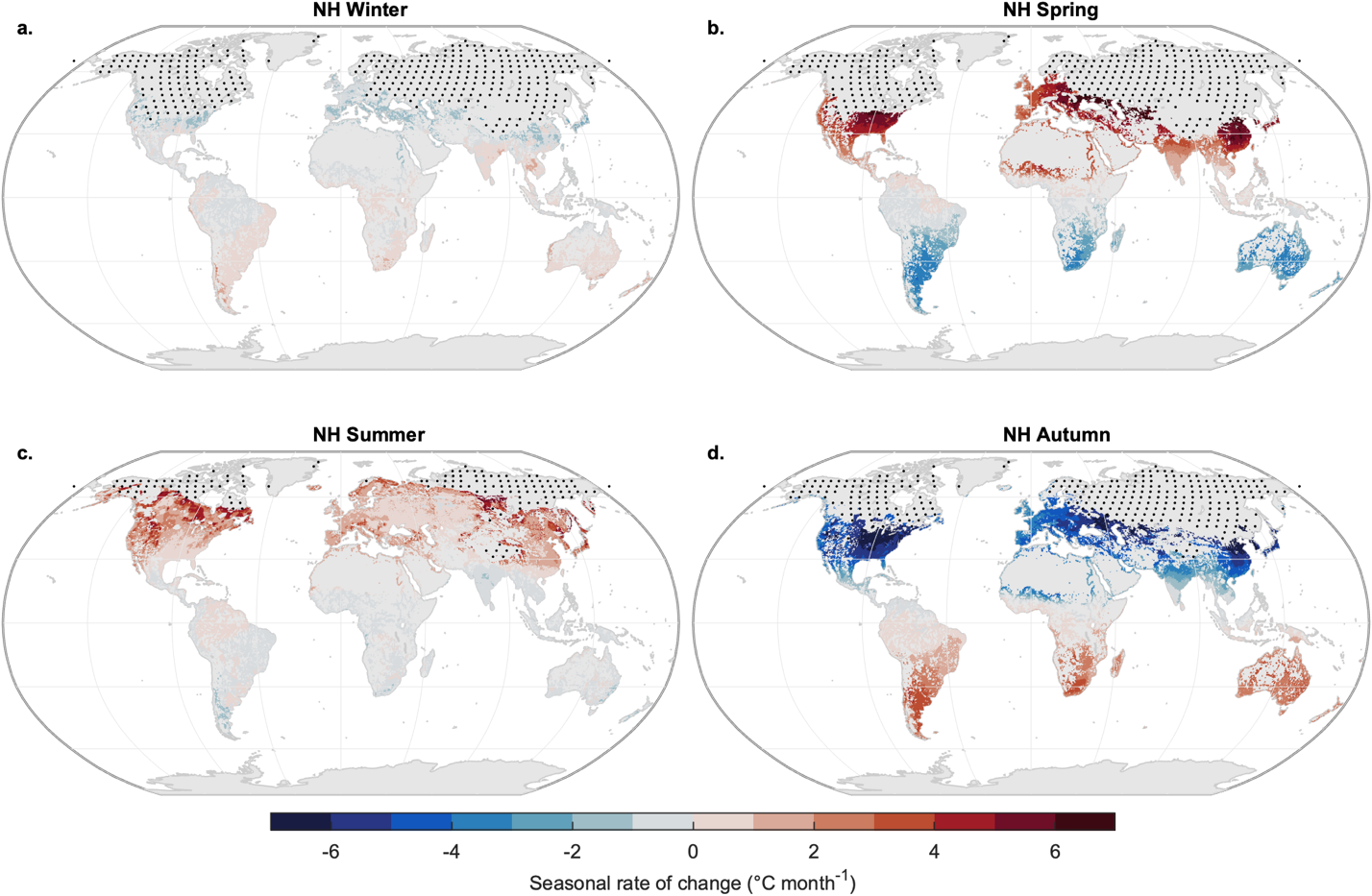
**

**Figure S4 |** Shown is the seasonal rate of change in lake surface water temperature during the historic to contemporary period (1980-2021) for (**a**) Winter (December-February in the Northern Hemisphere [NH] and June-August in Southern Hemisphere [SH]), (**b**) Spring (March-May in NH and September-November in the SH), (**c**) Summer (June-August in the NH and December-February in SH), and (**d**) Autumn (September-November in the NH and March-May in SH). Stipple markings represents regions that were not included in the analysis due to the presence of lake ice cover.

**
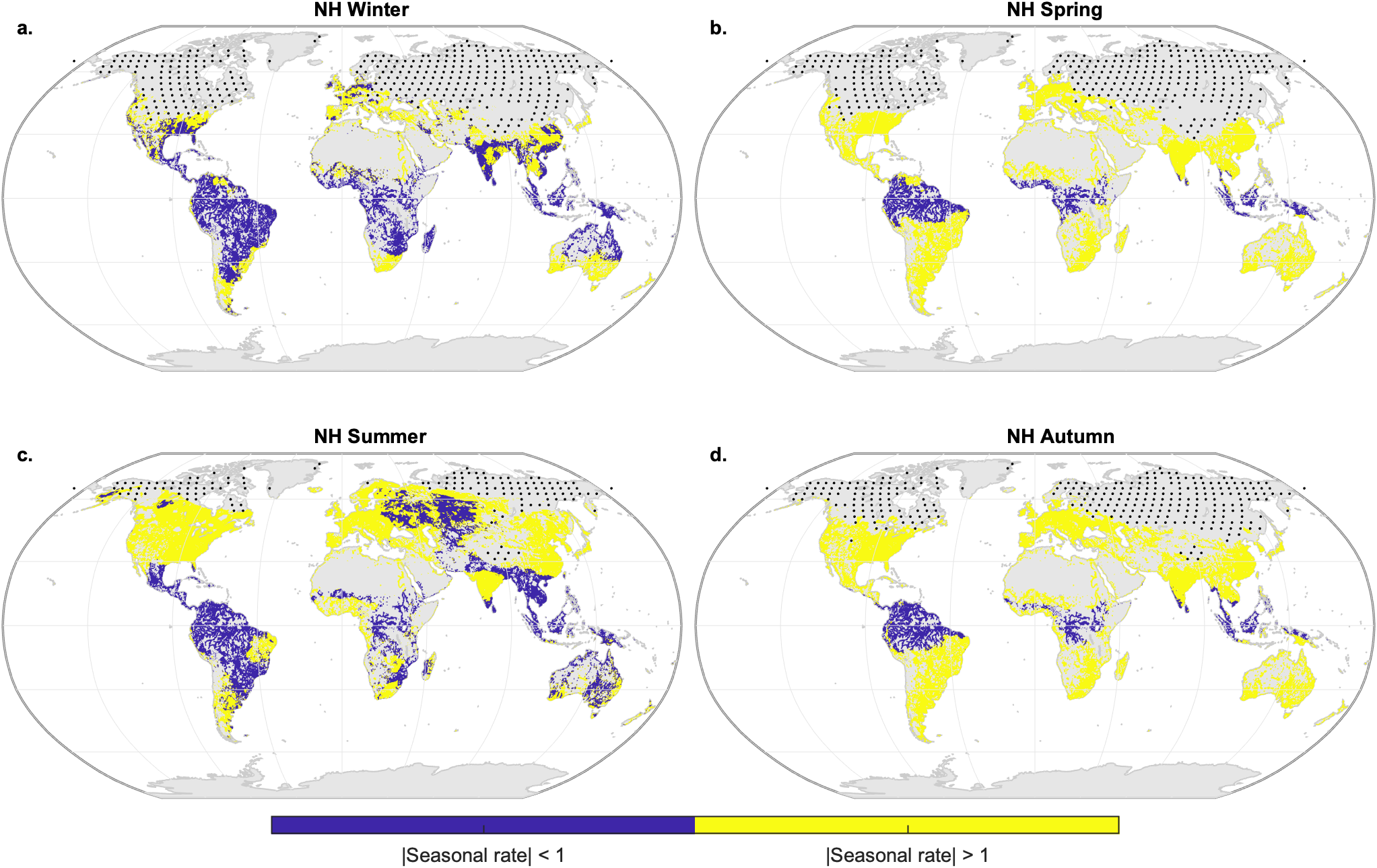
**

**Figure S5 |** Shown are lake regions that experience a minimal seasonal rate of change (< 0.5 °C month^-1^) in lake surface water temperature during the historic to contemporary period (1980-2021) in (**a**) Winter (December-February in the Northern Hemisphere [NH] and June-August in Southern Hemisphere [SH]), (**b**) Spring (March-May in NH and September-November in the SH), (**c**) Summer (June-August in the NH and December-February in SH), and (**d**) Autumn (September-November in the NH and March-May in SH). Stipple markings represents regions that were not included in the analysis due to the presence of lake ice cover. Lakes in blue are excluded from the investigation during the season of interest.

**
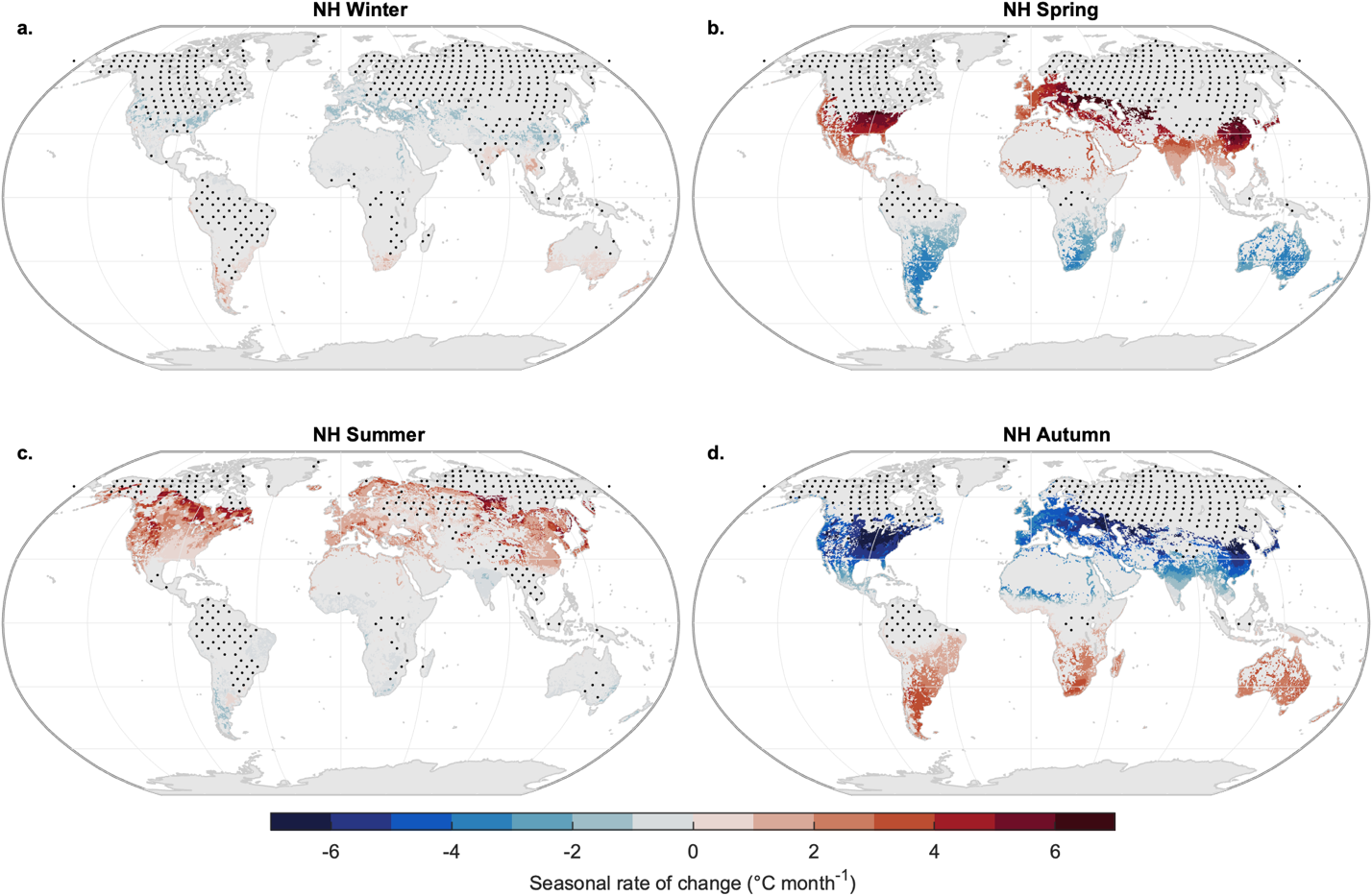
**

**Figure S6 |** Shown is the seasonal rate of change in lake surface water temperature during the historic to contemporary period (1980-2021) for (**a**) Winter (December-February in the Northern Hemisphere [NH] and June-August in Southern Hemisphere [SH]), (**b**) Spring (March-May in NH and September-November in the SH), (**c**) Summer (June-August in the NH and December-February in SH), and (**d**) Autumn (September-November in the NH and March-May in SH). Lakes that experienced ice cover or a minimal (< 0.5 °C month^-1^) seasonal rate of change in surface water temperature during the season of interest are omitted, and replaced by stipple markings.

**
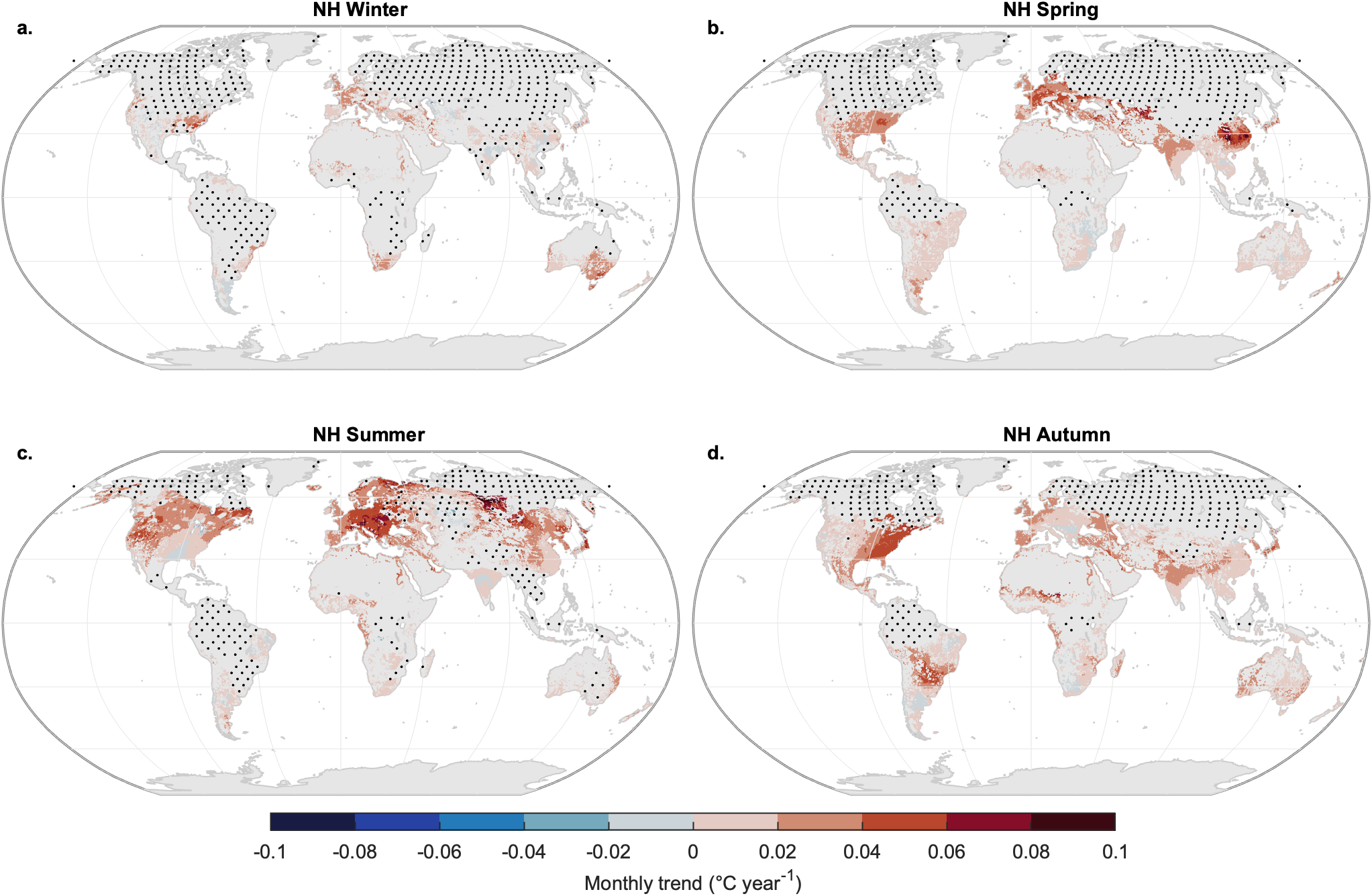
**

**Figure S7 |** Shown is the monthly trend in lake surface water temperature during the historic to contemporary period (1980-2021) for (**a**) Winter (January in the Northern Hemisphere [NH] and July in Southern Hemisphere [SH]), (**b**) Spring (April in NH and October in the SH), (**c**) Summer (July in the Northern Hemisphere and January in SH), and (**d**) Autumn (October in the NH and April in SH). Stipple markings represents regions that were not included in the analysis, either due to the presence of lake ice cover or when lakes experienced a minimal (< 0.5 °C month^-1^) seasonal rate of change in surface water temperature during the season of interest.

**
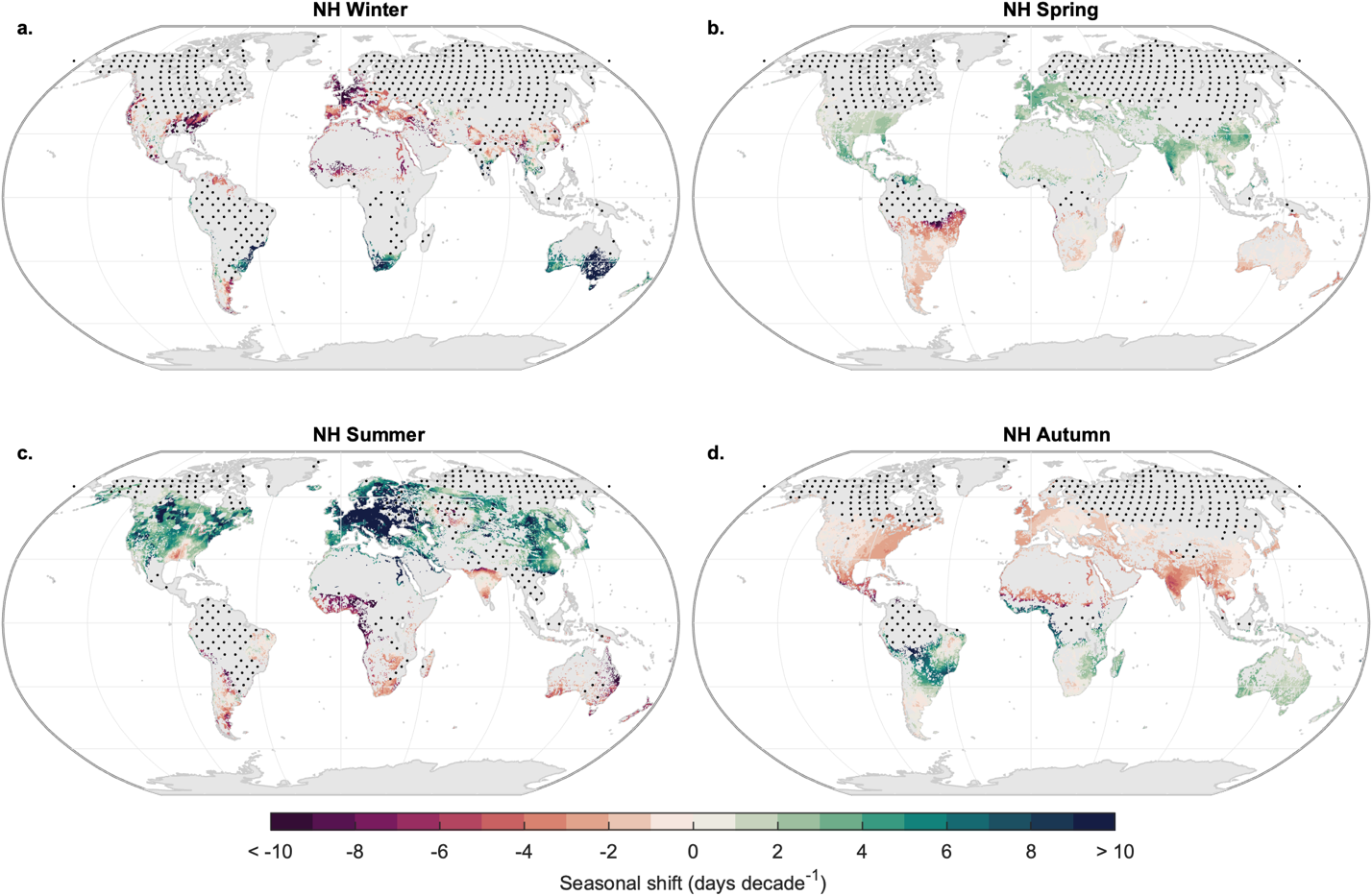
**

**Figure S8 |** Shown are the seasonal shifts (days decade^-1^) in the timing of (**a**) Winter (December-February in the Northern Hemisphere [NH] and June-August in Southern Hemisphere [SH]), (**b**) Spring (March-May in NH and September-November in the SH), (**c**) Summer (June-August in the NH and December-February in SH), and (**d**) Autumn (September-November in the NH and March-May in SH) during the historic to contemporary period (1980-2021). Positive and negative values indicate when the timing of a season has advanced or been delayed, respectively. Stipple markings represents regions that were not included in the analysis, either due to the presence of lake ice cover or when lakes experienced a minimal (< 0.5 °C month^-1^) seasonal rate of change in surface water temperature during the season of interest.


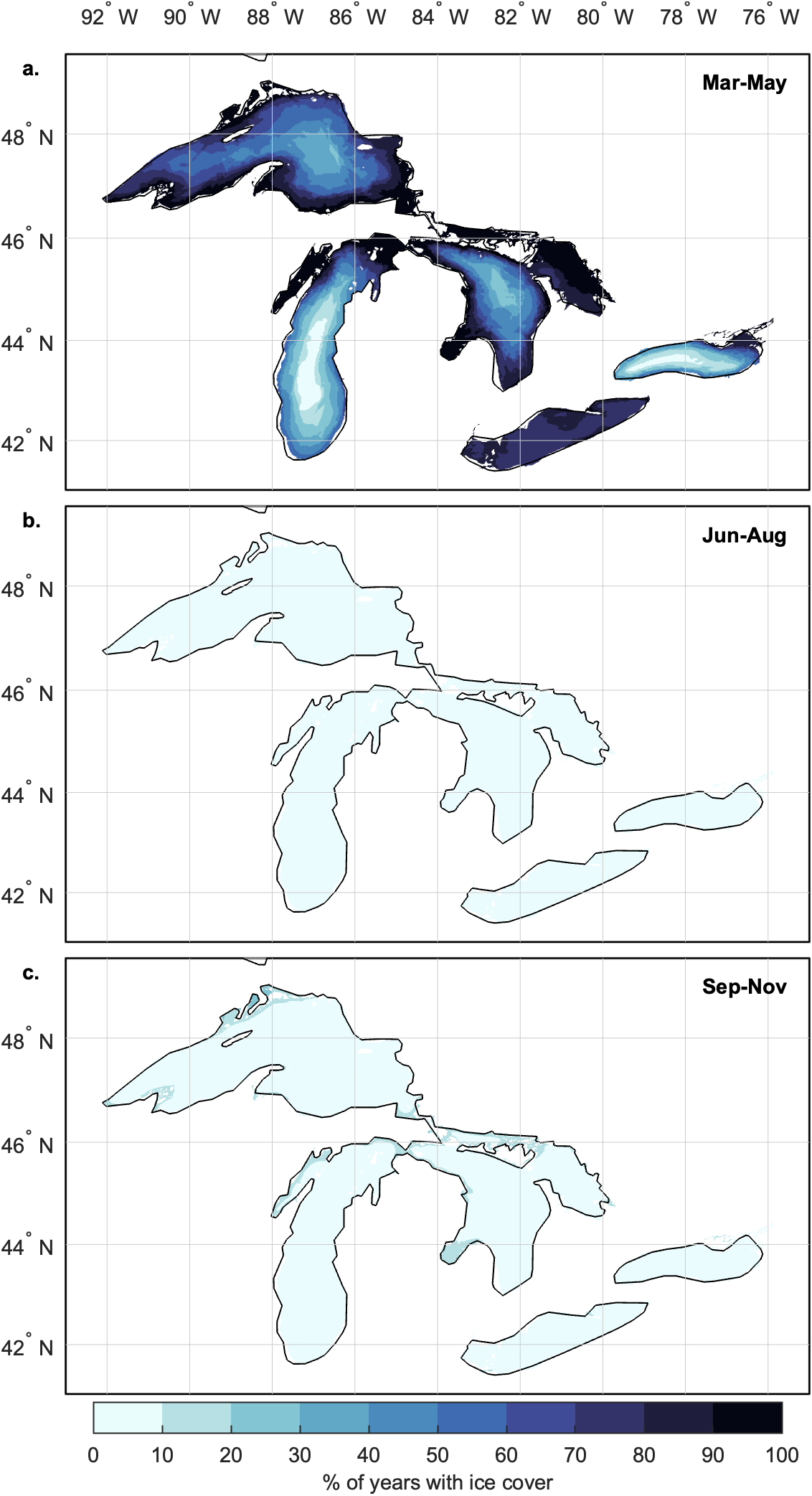


**Figure S9 |** Shown is the percentage of years (1995-2021) where the North American Great Lakes experience ice cover during (**a**) Spring (Mar-May), (**b**) Summer (June-Aug), and (**c**) Autumn (Sep-Nov).


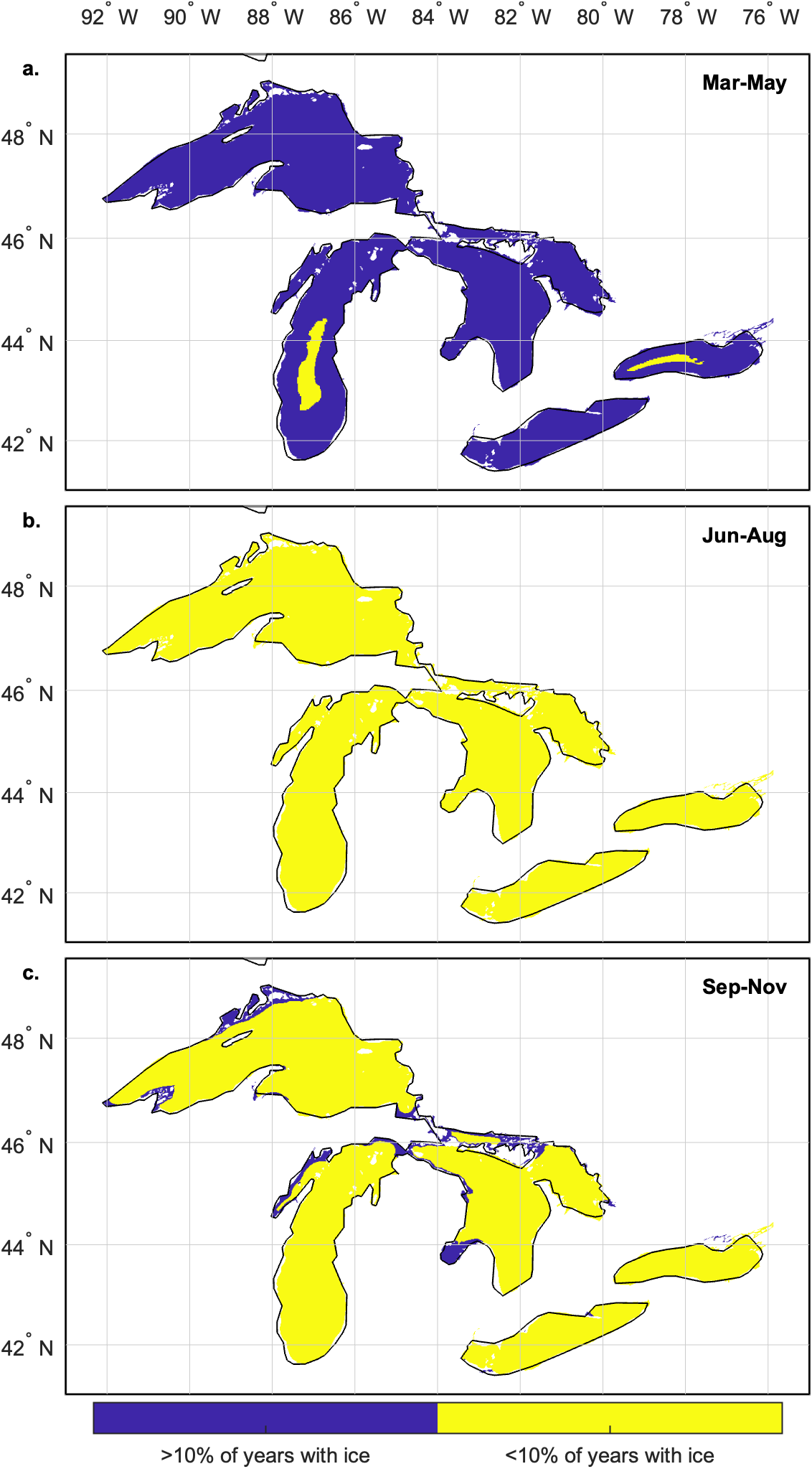


**Figure S10 |** Shown are regions within the Great Lakes that experience ice cover for more (blue) or less (yellow) than 10% of all years during the satellite data-taking period (1995-2021) during (**a**) Spring (Mar-May), (**b**) Summer (June-Aug), and (**c**) Autumn (Sep-Nov). Within-lake regions shown in blue are excluded from the investigation during the season of interest.


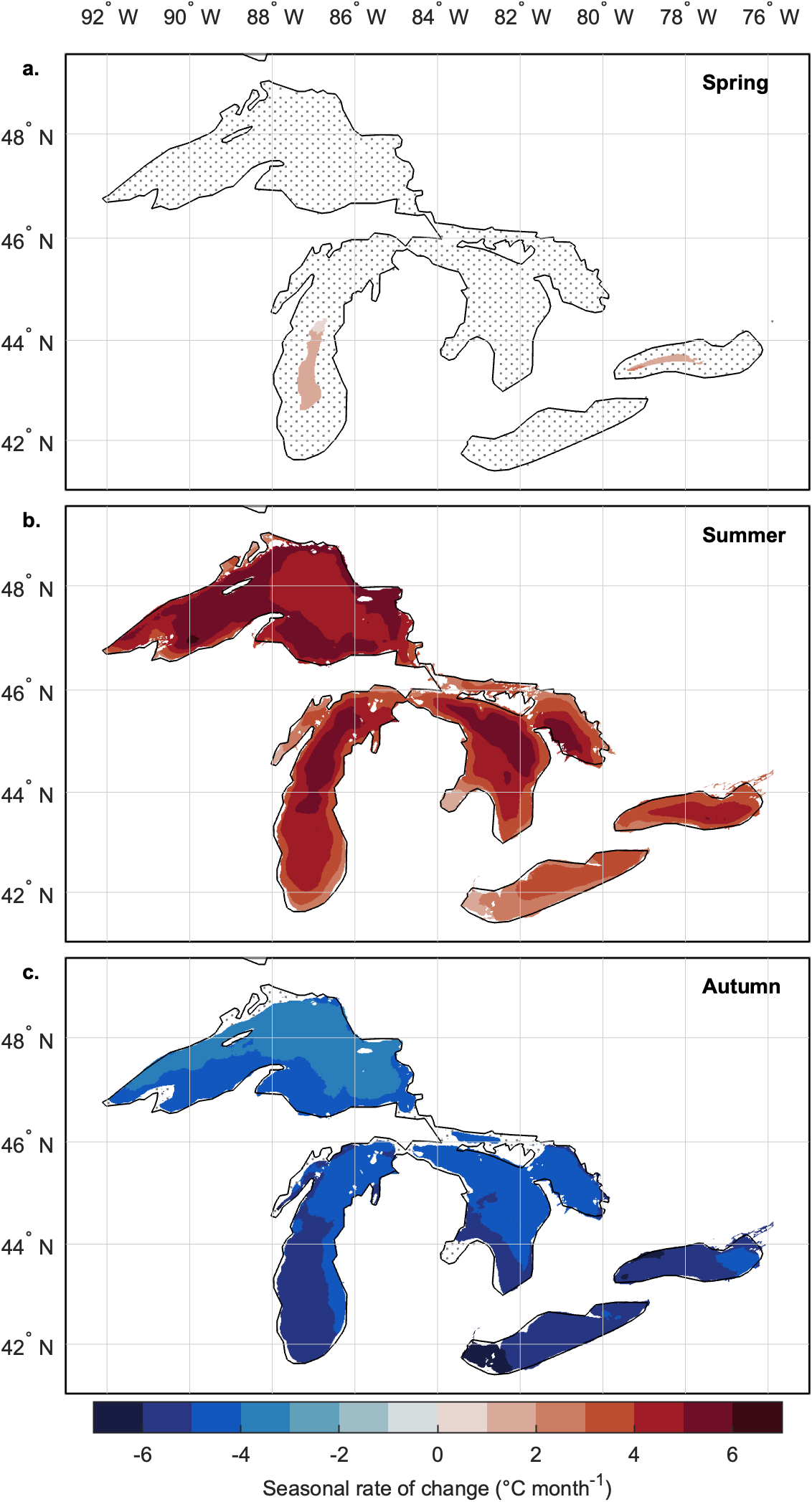


**Figure S11 |** Shown is the seasonal rate of change in lake surface water temperature during the satellite data-taking period (1995-2021) within the Great Lakes during (**a**) Spring (Mar-May), (**b**) Summer (June-Aug), and (**c**) Autumn (Sep-Nov). Stipple markings represents regions that were not included in the analysis due to the presence of lake ice cover during the season of interest.

**
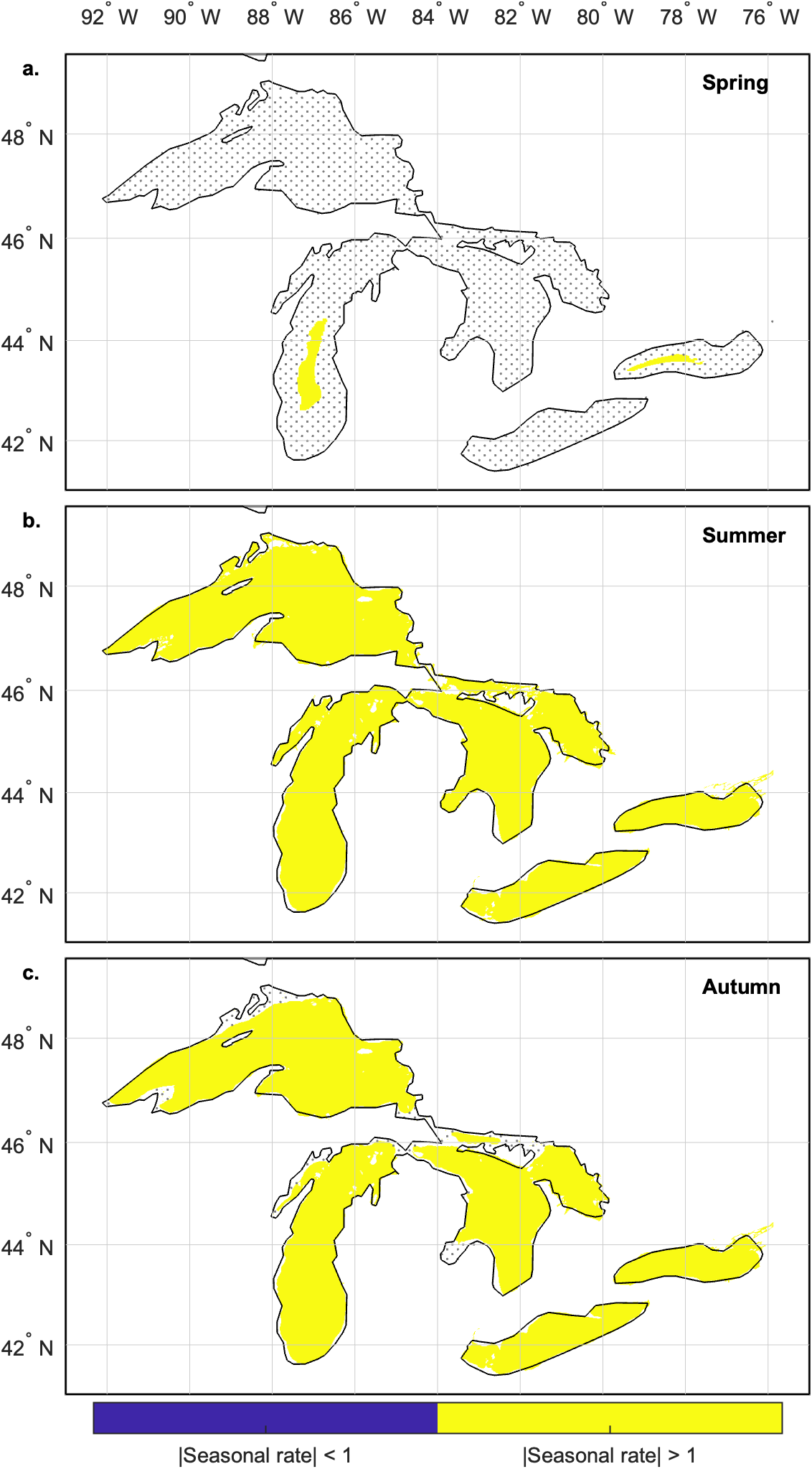
**

**Figure S12 |** Shown are the within-lake regions that experience a minimal seasonal rate of change (< 0.5 °C month^-1^) in lake surface water temperature during the satellite data-taking period (1995-2021) in the Great Lakes during (**a**) Spring (Mar-May), (**b**) Summer (June-Aug), and (**c**) Autumn (Sep-Nov). Stipple markings represents regions that were not included in the analysis due to the presence of lake ice cover. Within-lake regions in blue (if any) are excluded from the investigation during the season of interest.

**
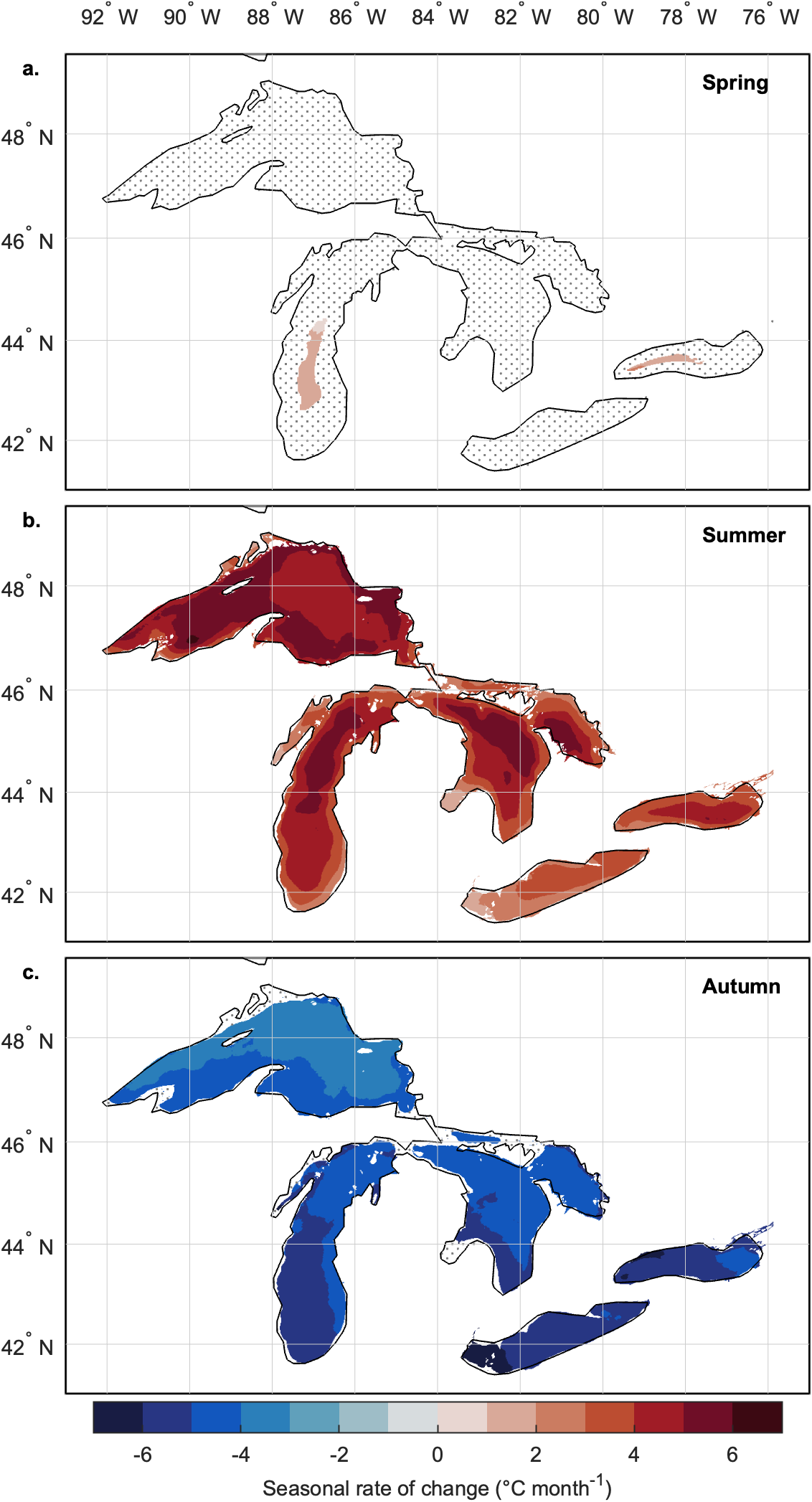
**

**Figure S13 |** Shown is the seasonal rate of change in lake surface water temperature during the satellite data-taking period (1995-2021) within the Great Lakes during (**a**) Spring (Mar-May), (**b**) Summer (June-Aug), and (**c**) Autumn (Sep-Nov). Within-lake regions that experienced ice cover or a minimal (< 0.5 °C month^-1^) seasonal rate of change in surface water temperature during the season of interest are omitted and replaced by stipple markings.

**
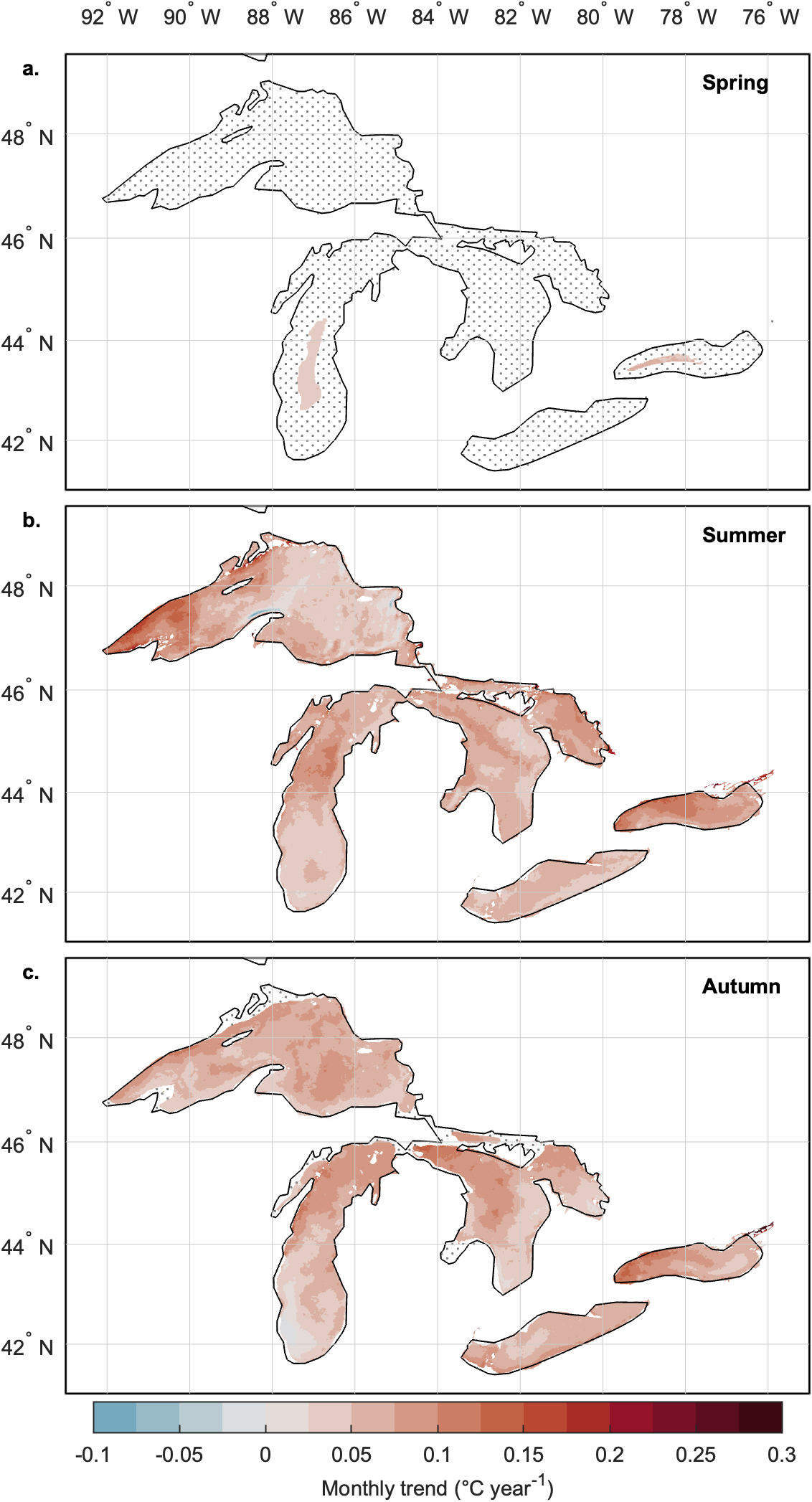
**

**Figure S14 |** Shown is the monthly trend in lake surface water temperature during the satellite data-taking period (1995-2021) in the Great Lakes for (**a**) Spring (Mar-May), (**b**) Summer (June-Aug), and (**c**) Autumn (Sep-Nov). Stipple markings represents regions that were not included in the analysis, either due to the presence of lake ice cover or when lakes experienced a minimal (< 0.5 °C month^-1^) seasonal rate of change in surface water temperature during the season of interest.

**
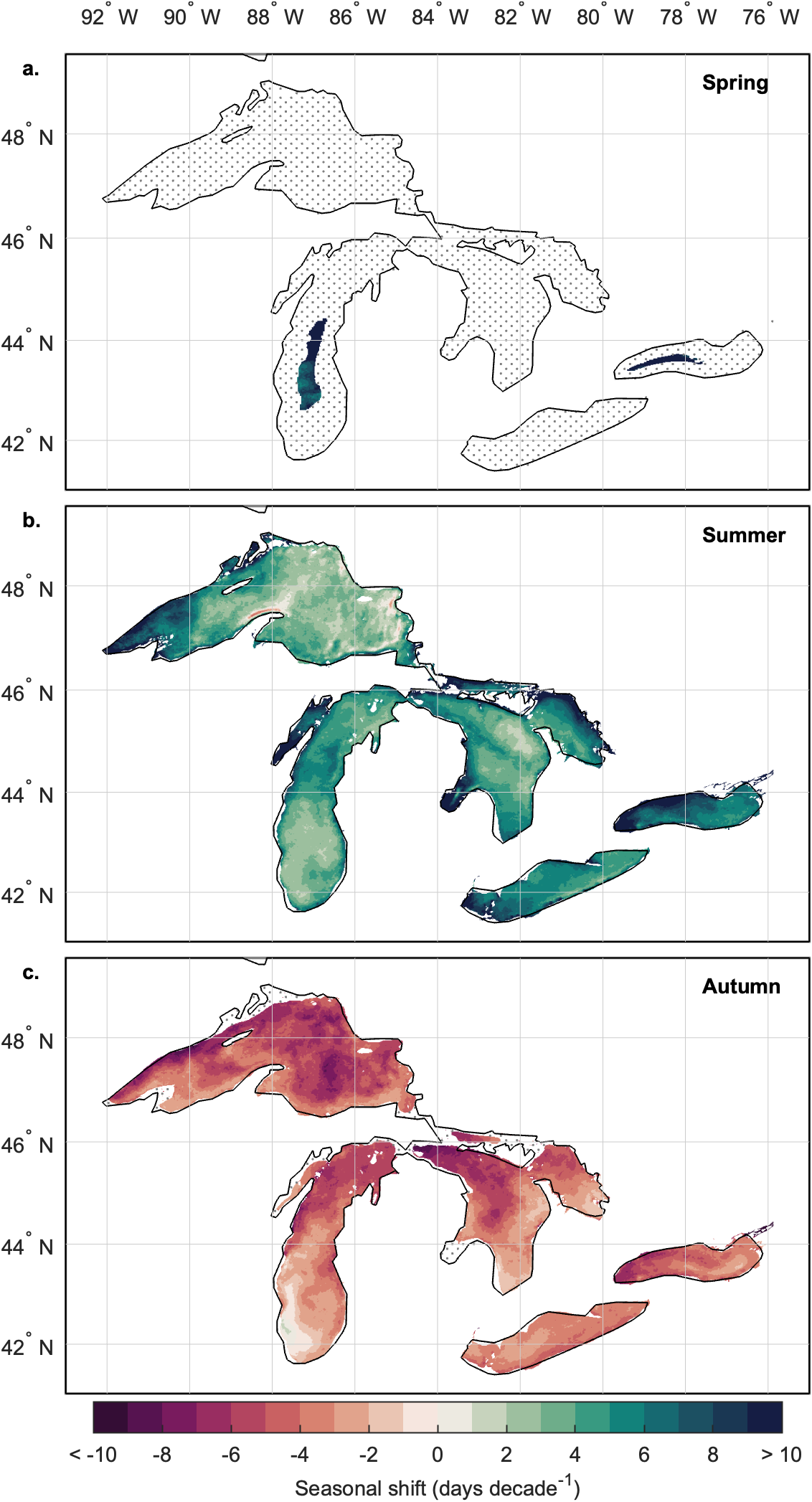
**

**Figure S15 |** Shown are the seasonal shifts (days decade^-1^) in the timing of (**a**) Spring (Mar-May), (**b**) Summer (June-Aug), and (**c**) Autumn (Sep-Nov) during the satellite data-taking period (1995-2021) in the Great Lakes. Positive and negative values indicate when the timing of a season has advanced or been delayed, respectively. Stipple markings represents regions that were not included in the analysis, either due to the presence of lake ice cover or when lakes experienced a minimal (< 0.5 °C month^-1^) seasonal rate of change in surface water temperature during the season of interest.

**
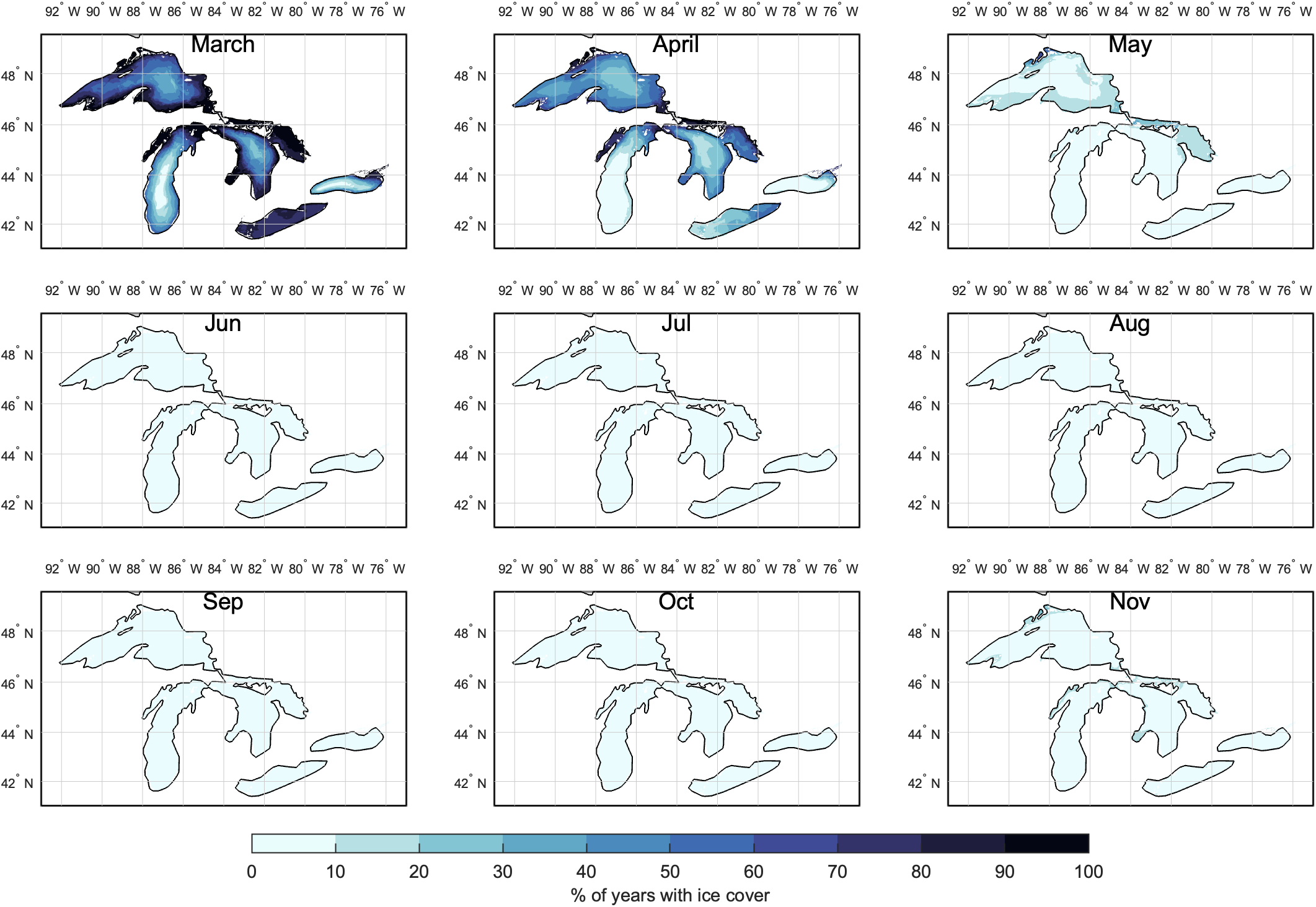
**

**Figure S16 |** Shown is the percentage of years (1995-2021) where the North American Great Lakes experience ice cover during each month from March to November.

**
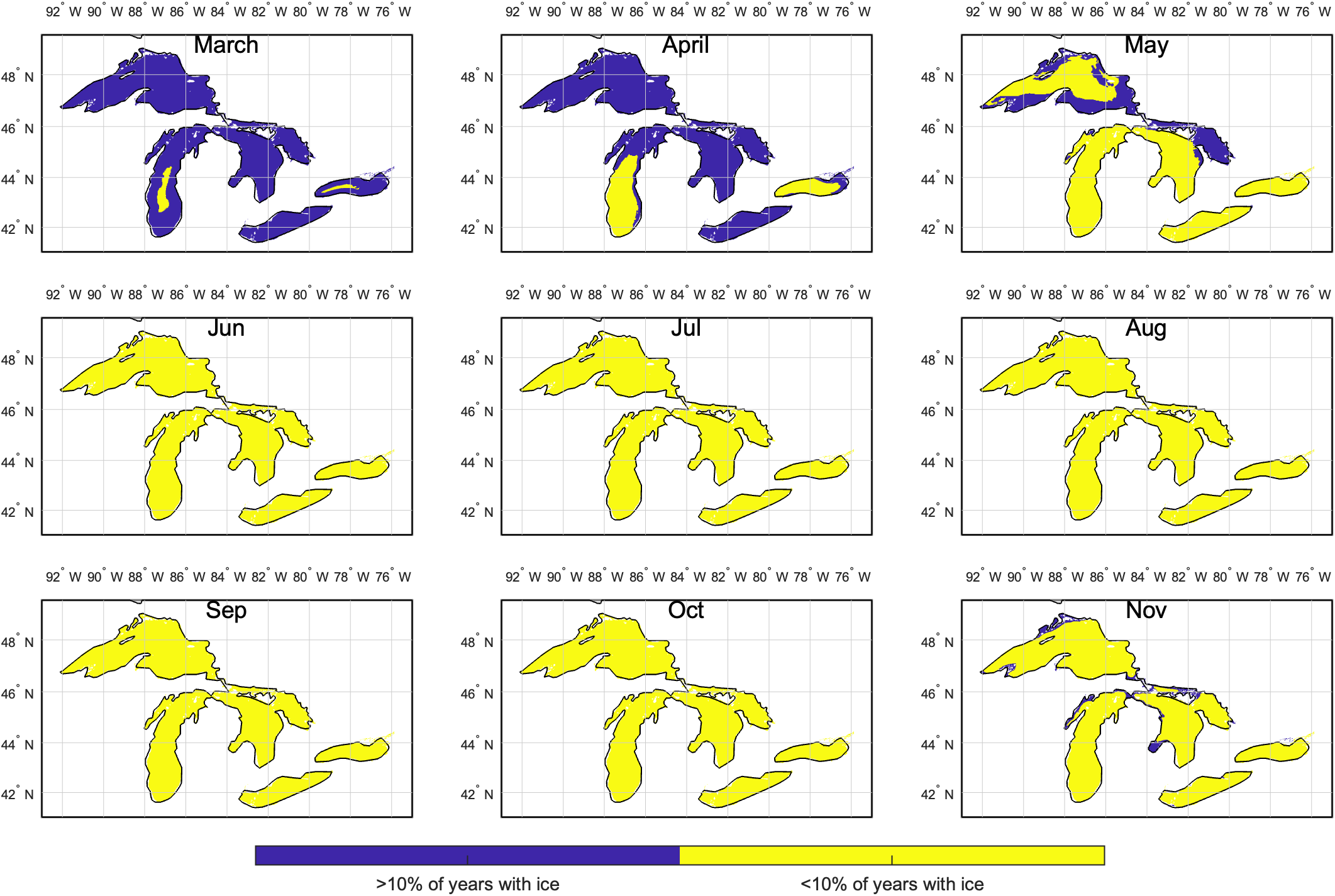
**

**Figure S17 |** Shown are regions within the Great Lakes that experience ice cover for more (blue) or less (yellow) than 10% of all years during the satellite data-taking period (1995-2021) for each month from March to November. Within-lake regions shown in blue are excluded from the investigation during the season of interest.

**
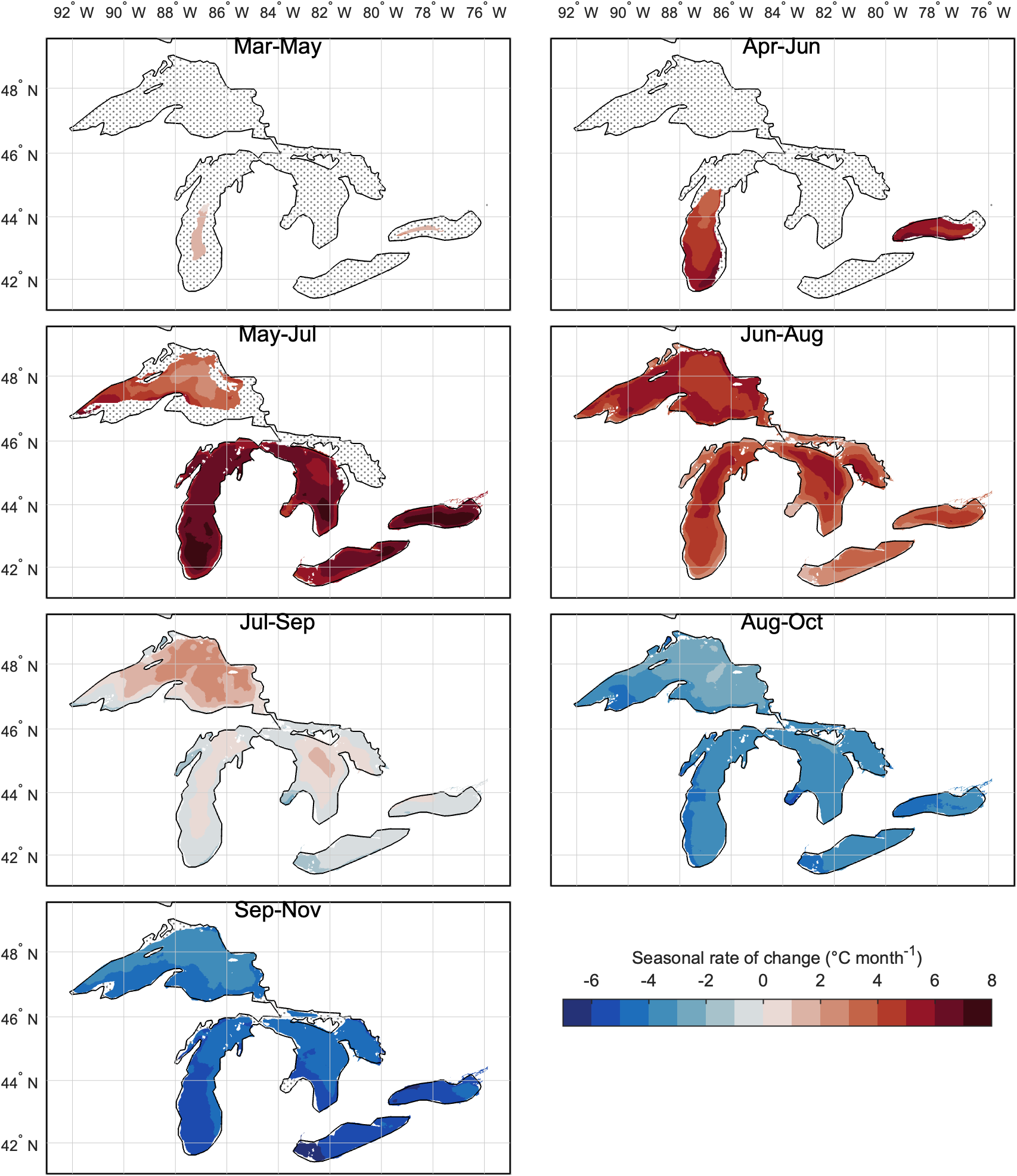
**

**Figure S18 |** Shown is the seasonal rate of change in lake surface water temperature during the satellite data-taking period (1995-2021) within the Great Lakes for each three-month moving window from March to November. Stipple markings represents regions that were not included in the analysis due to the presence of lake ice cover during the season of interest.

**
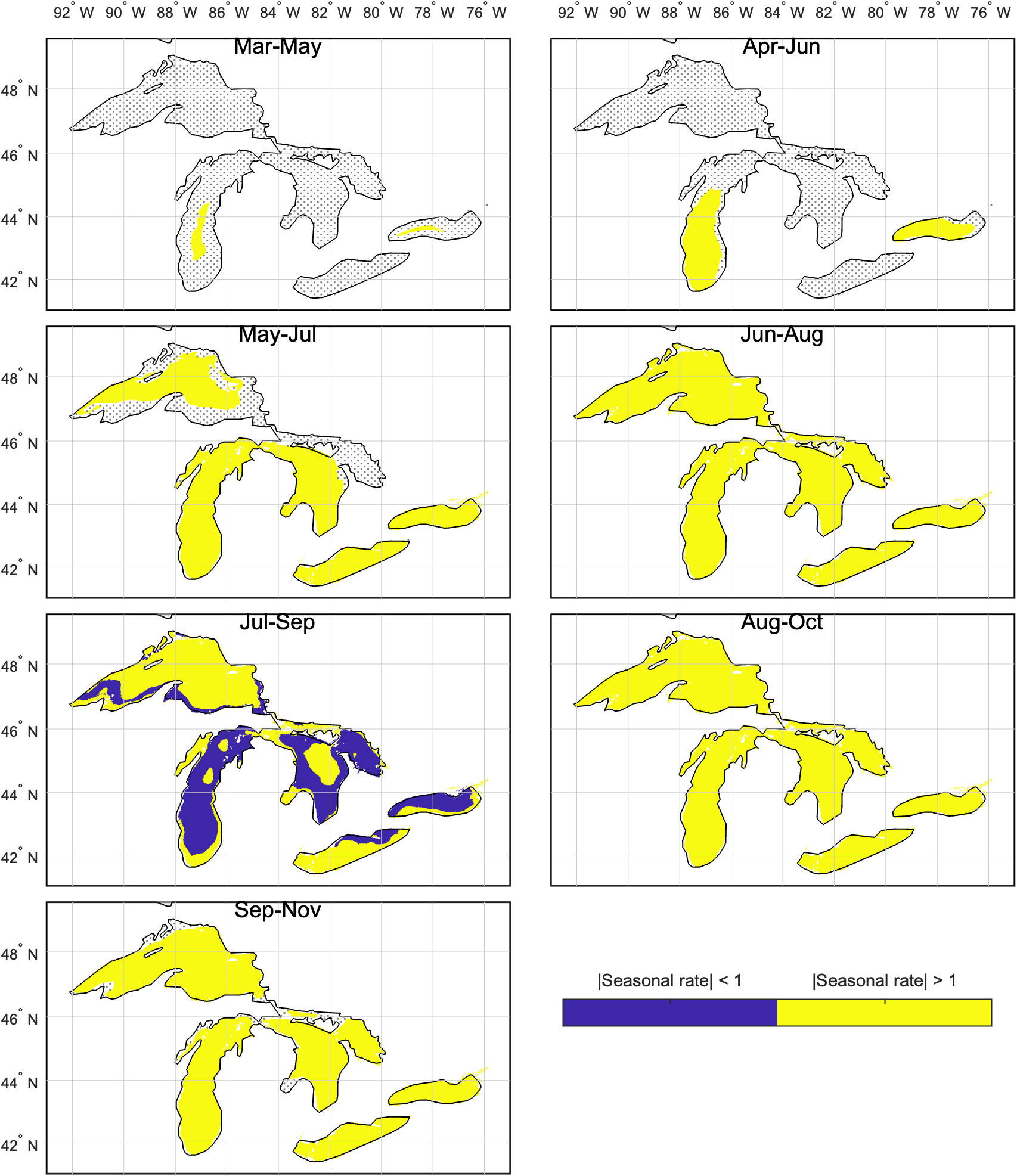
**

**Figure S19 |** Shown are the within-lake regions that experience a minimal seasonal rate of change (< 0.5 °C month^-1^) in lake surface water temperature during the satellite data-taking period (1995-2021) within the Great Lakes for each three-month moving window from March to November. Stipple markings represents regions that were not included in the analysis due to the presence of lake ice cover. Within-lake regions in blue (if any) are excluded from the investigation during the season of interest.

**
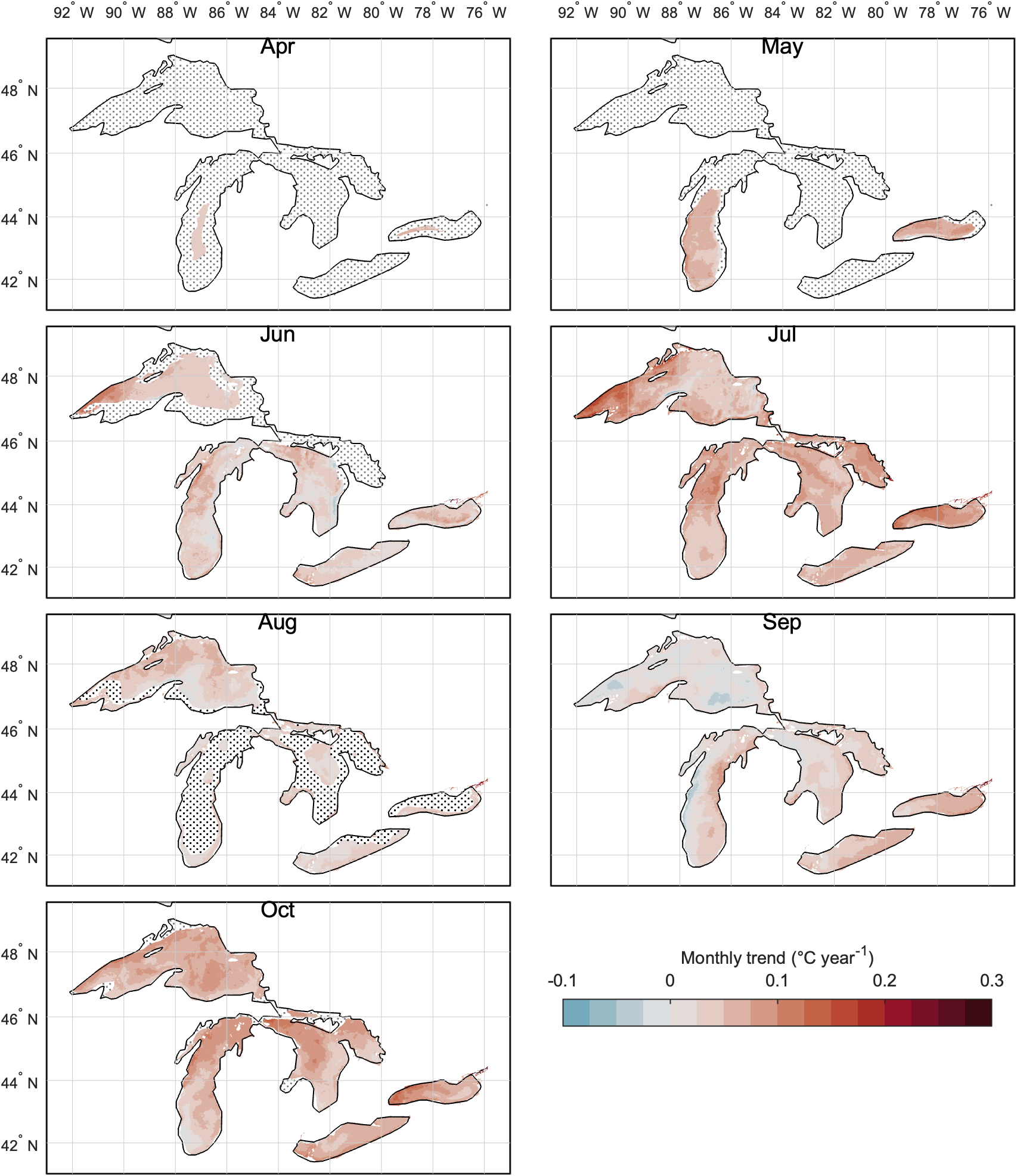
**

**Figure S20 |** Shown is the monthly trend in lake surface water temperature during the satellite data-taking period (1995-2021) within the Great Lakes for each three-month moving window from March to November. Stipple markings represents regions that were not included in the analysis, either due to the presence of lake ice cover or when lakes experienced a minimal (< 0.5 °C month^-1^) seasonal rate of change in surface water temperature during the season of interest.

**
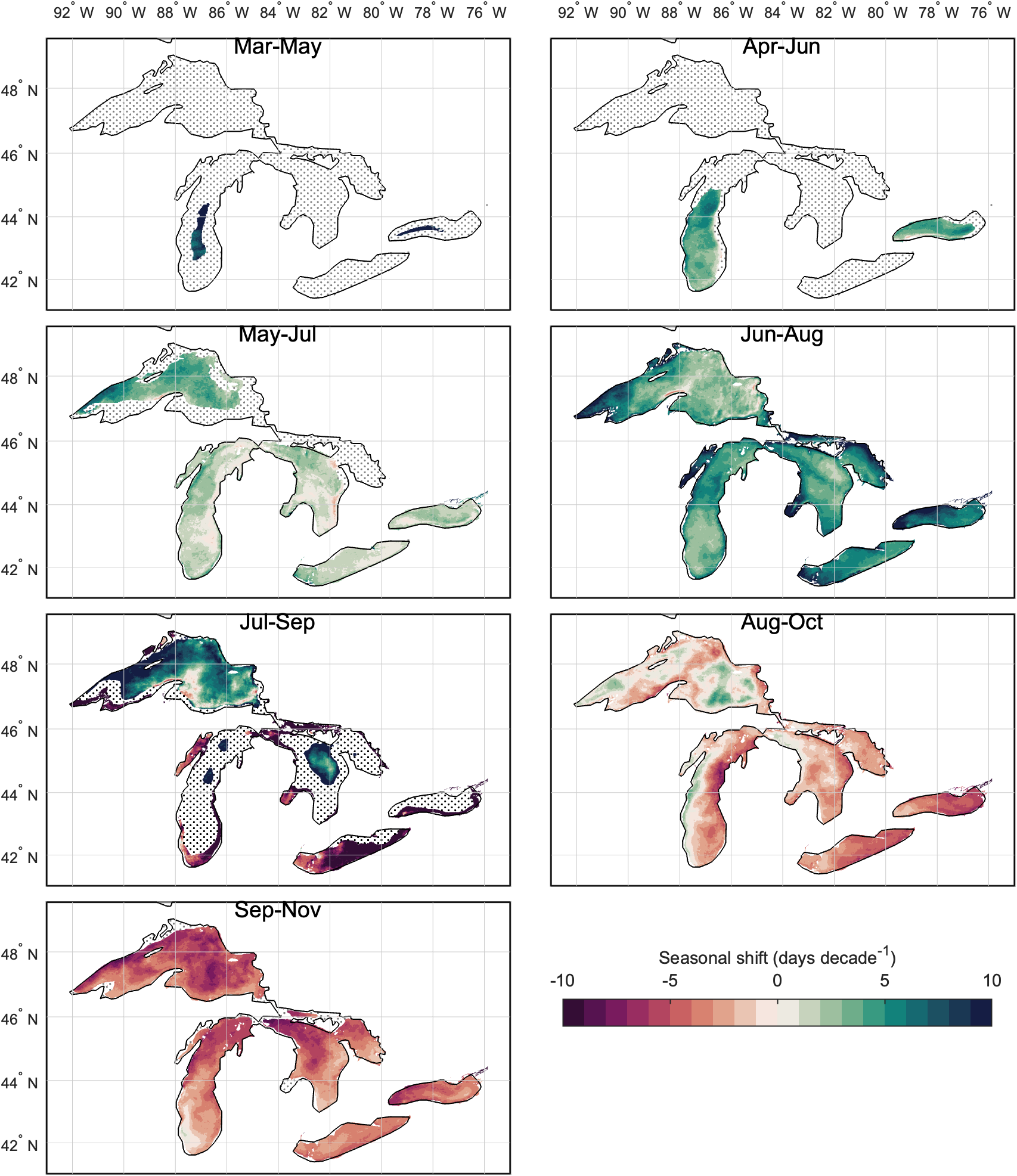
**

**Figure S21 |** Shown are the seasonal shifts (days decade^-1^) in the timing of the seasons, defined according to a three-month moving window from March to November. Positive and negative values indicate when the timing of a season has advanced or been delayed, respectively. Stipple markings represents regions that were not included in the analysis, either due to the presence of lake ice cover or when lakes experienced a minimal (< 0.5 °C month^-1^) seasonal rate of change in surface water temperature during the season of interest.

**
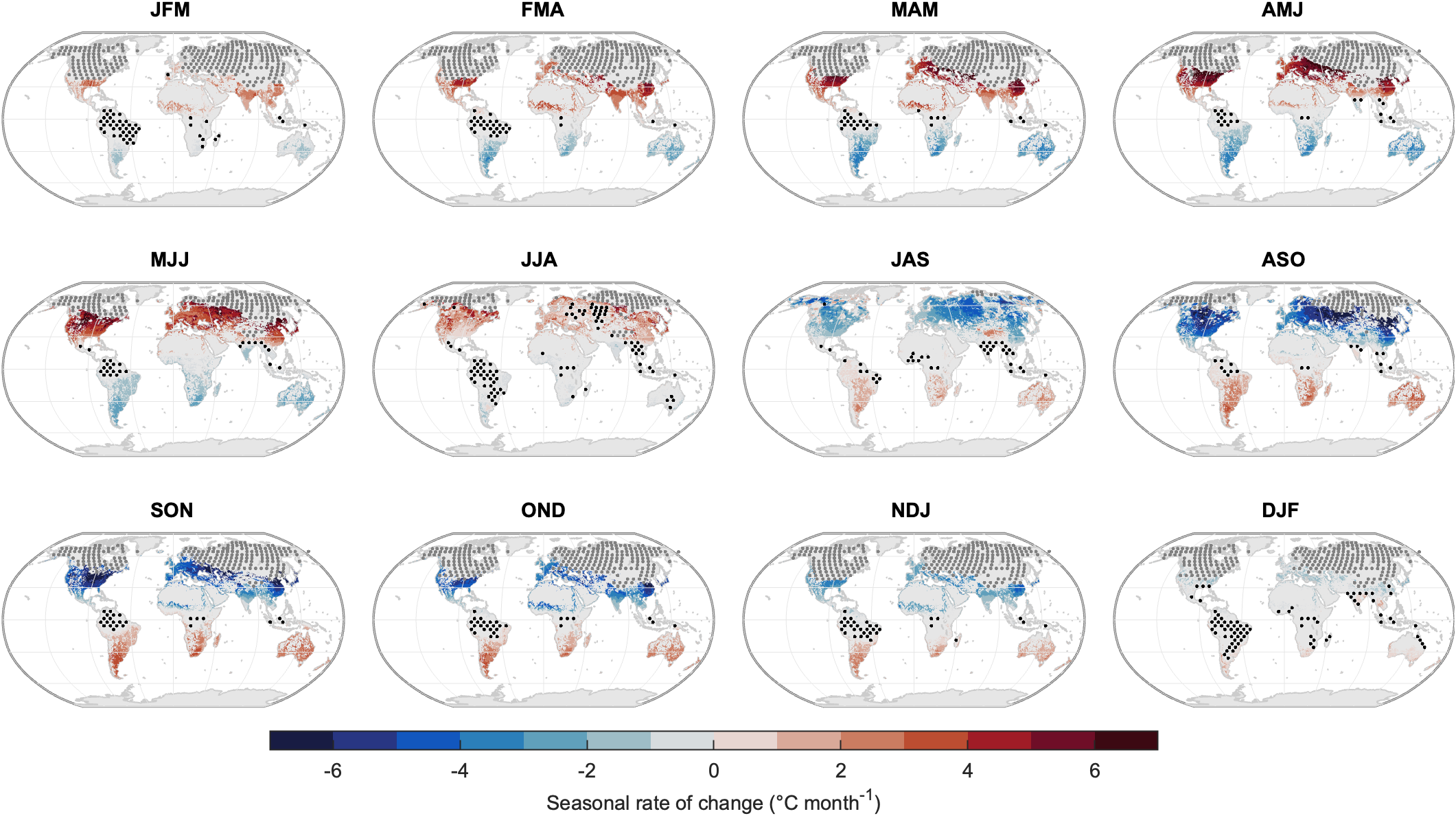
**

**Figure S22 |** Shown is the seasonal rate of change in lake surface water temperature during the historic to contemporary period (1980-2021). Here, the seasons are defined according to a three-month moving window. Stipple markings represents regions that were not included in the analysis, either due to the presence of lake ice cover or when lakes experienced a minimal (< 0.5 °C month^-1^) seasonal rate of change in surface water temperature during the season of interest.

**
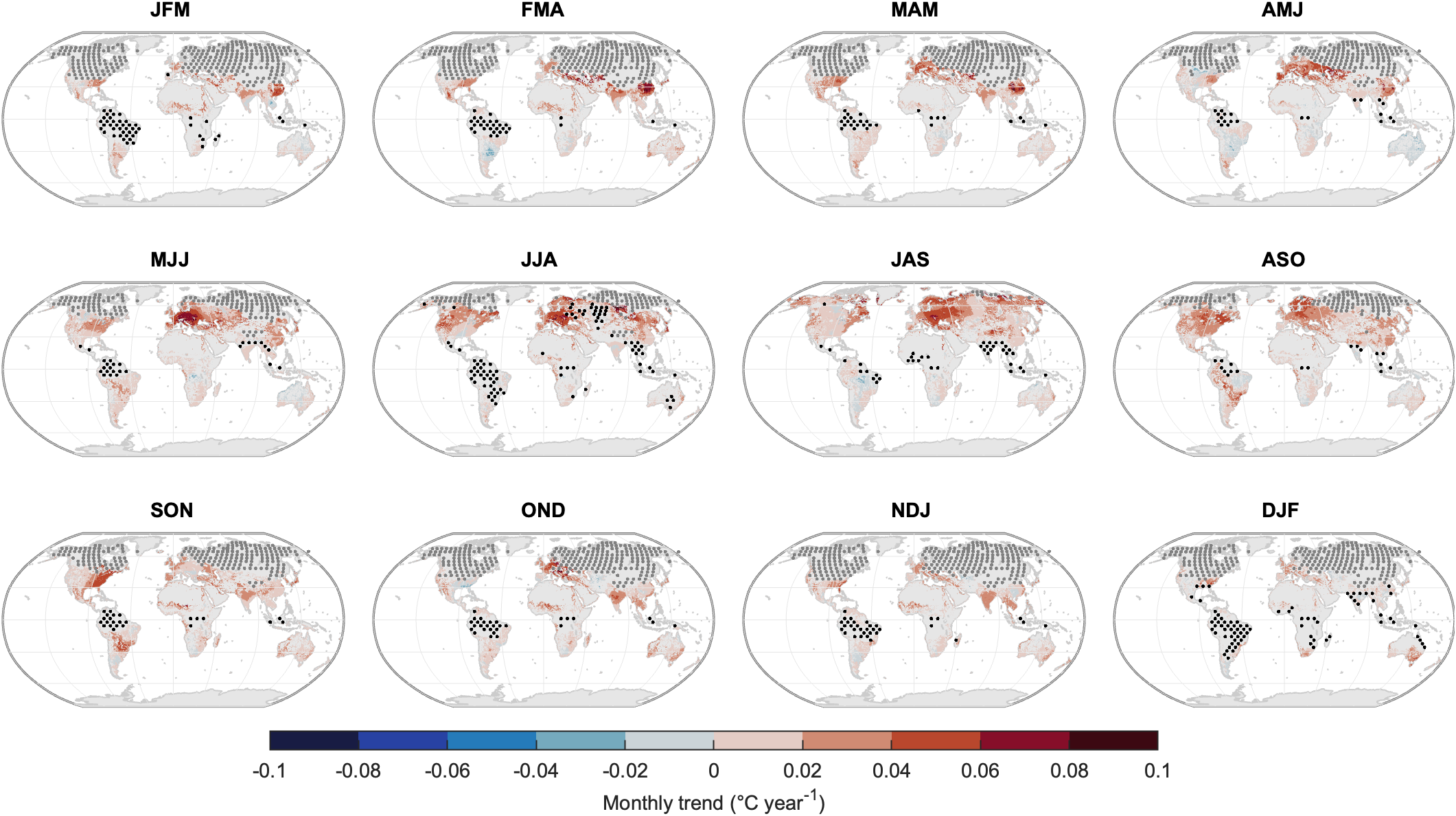
**

**Figure S23 |** Shown is the monthly trend in lake surface water temperature during the historic to contemporary period (1980-2021). Here, the seasons are defined according to a three-month moving window. Stipple markings represents regions that were not included in the analysis, either due to the presence of lake ice cover or when lakes experienced a minimal (< 0.5 °C month^-1^) seasonal rate of change in surface water temperature during the season of interest.

**
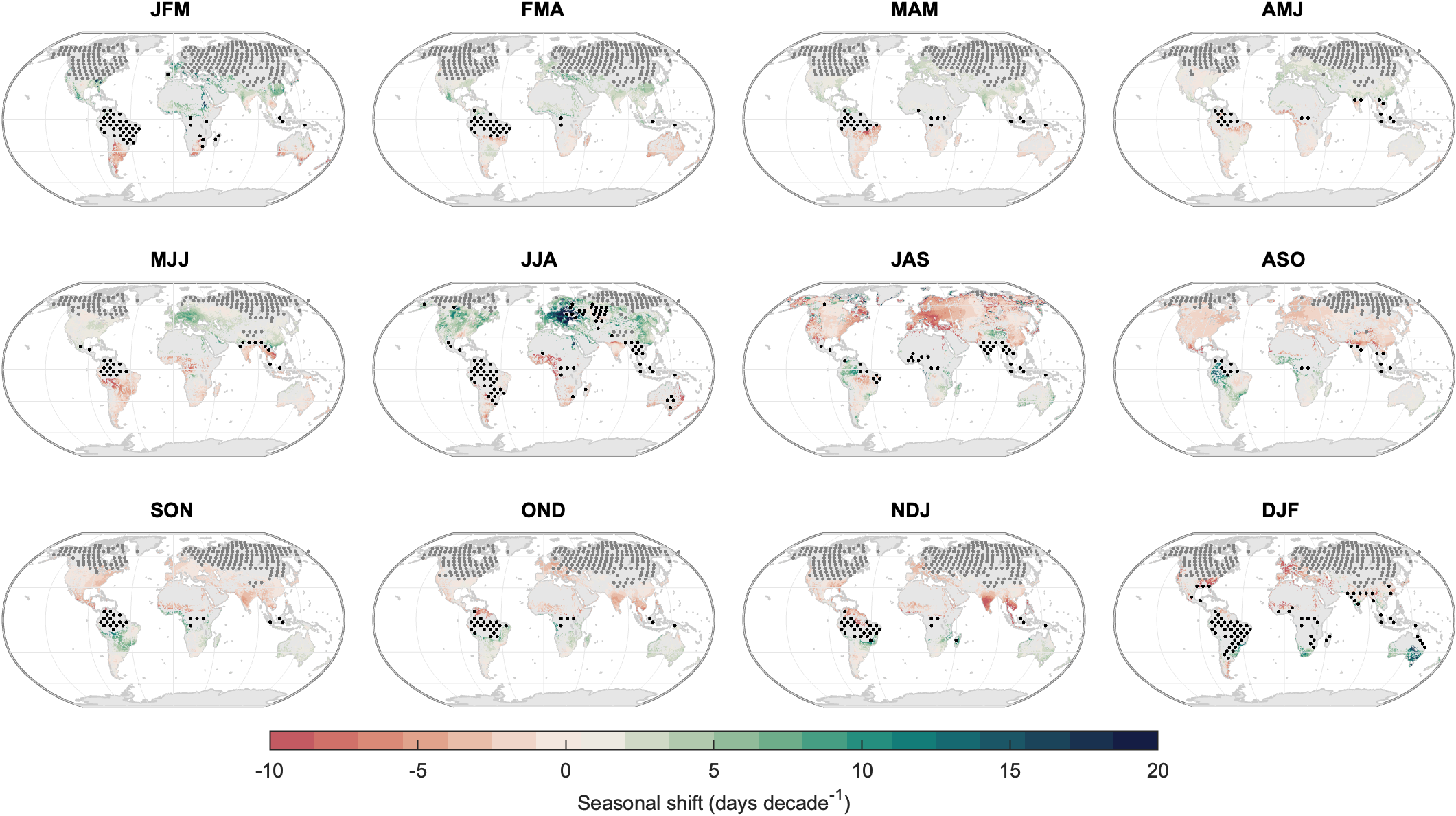
**

**Figure S24 |** Shown are the seasonal shifts (days decade^-1^) in the timing of seasonal temperatures during the historic to contemporary period (1980-2021). Here, the seasons are defined according to a three-month moving window. Positive and negative values indicate when the timing of a season has advanced or been delayed, respectively. Stipple markings represents regions that were not included in the analysis, either due to the presence of lake ice cover or when lakes experienced a minimal (< 0.5 °C month^-1^) seasonal rate of change in surface water temperature during the season of interest.


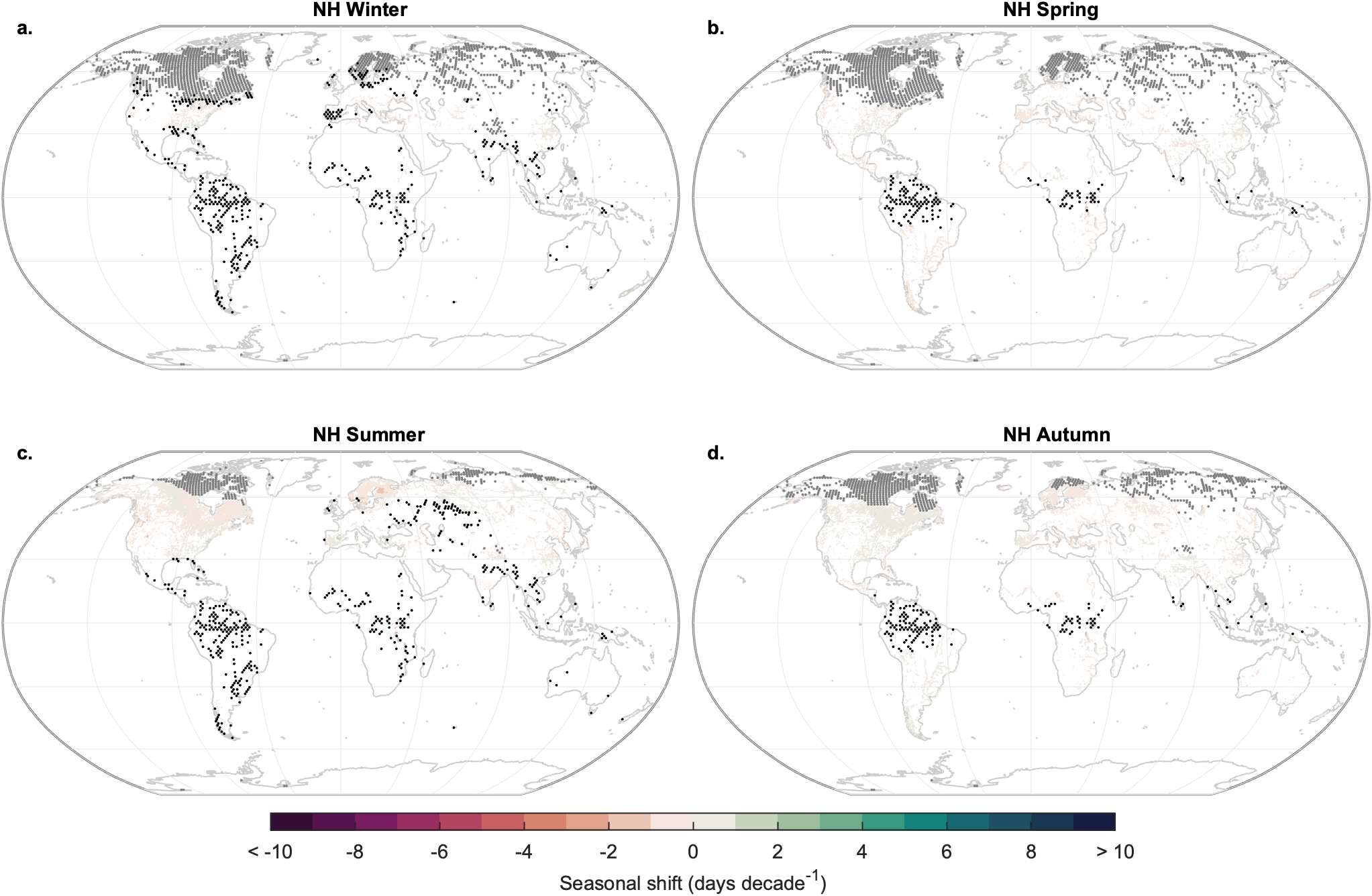
 **Figure S25 |** Shown are future (2021-2099) seasonal shifts (days decade^-1^) in the timing of **(a)** winter (December-February in the Northern Hemisphere [NH] and June-August in Southern Hemisphere [SH]), (**b**) spring (March-May in NH and September-November in the SH), **(c)** summer (June-August in the NH and December-February in SH), and **(d)** autumn (September-November in the NH and March-May in SH) in lakes worldwide under pre-industrial climate forcing. Positive and negative values indicate when the timing of a season is projected to advance or be delayed, respectively. Stipple markings represents regions that were not included in the analysis, either due to the presence of lake ice cover or when lakes experienced a minimal (< 0.5 °C month^-1^) seasonal rate of change in surface water temperature during the season of interest.


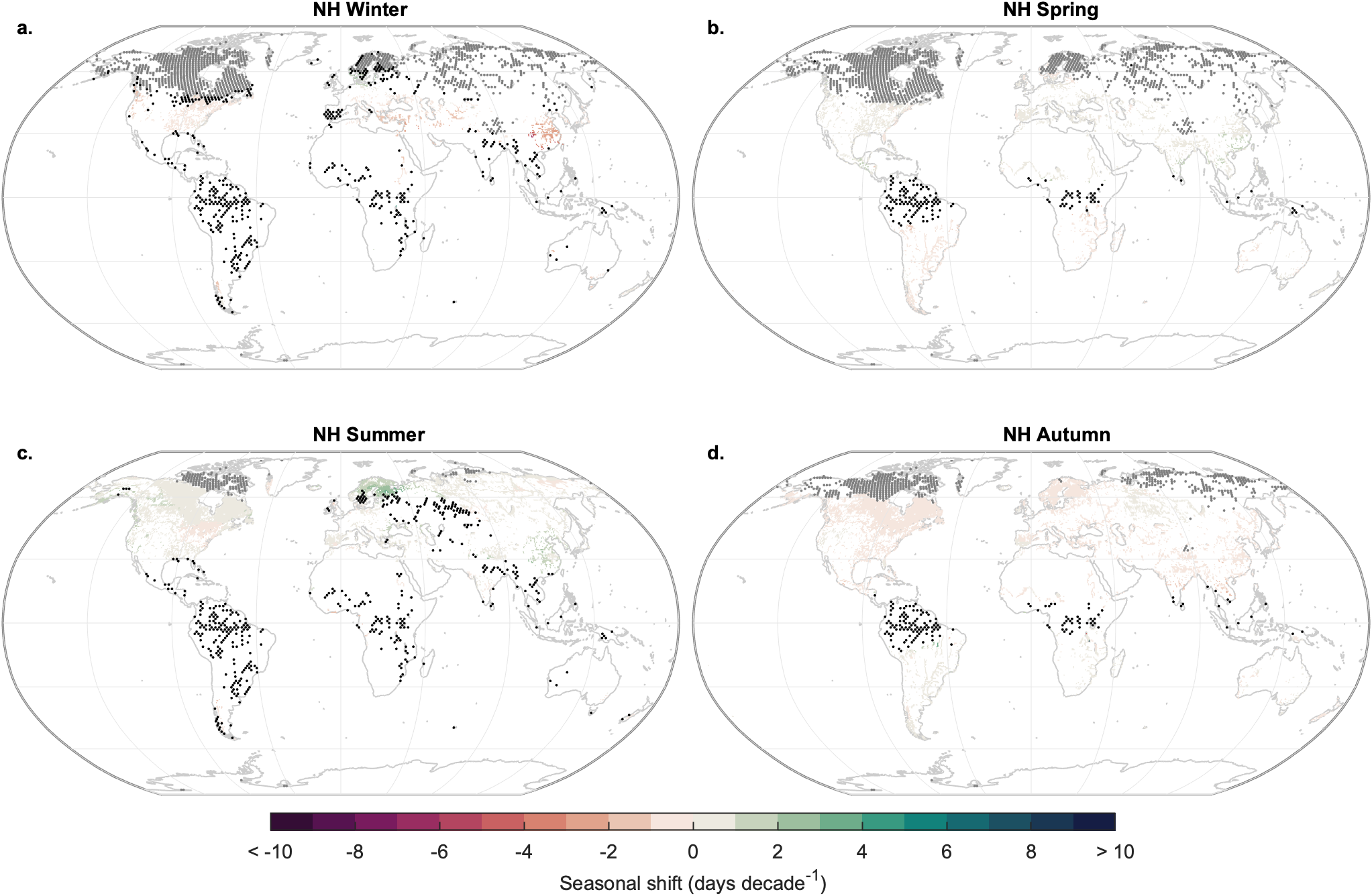
 **Figure S26 |** Shown are future (2021-2099) seasonal shifts (days decade^-1^) in the timing of **(a)** winter (December-February in the Northern Hemisphere [NH] and June-August in Southern Hemisphere [SH]), (**b**) spring (March-May in NH and September-November in the SH), **(c)** summer (June-August in the NH and December-February in SH), and **(d)** autumn (September-November in the NH and March-May in SH) in lakes worldwide under Representative Concentration Pathway (RCP) 2.6. Positive and negative values indicate when the timing of a season is projected to advance or be delayed, respectively. Stipple markings represents regions that were not included in the analysis, either due to the presence of lake ice cover or when lakes experienced a minimal (< 0.5 °C month^-1^) seasonal rate of change in surface water temperature during the season of interest.


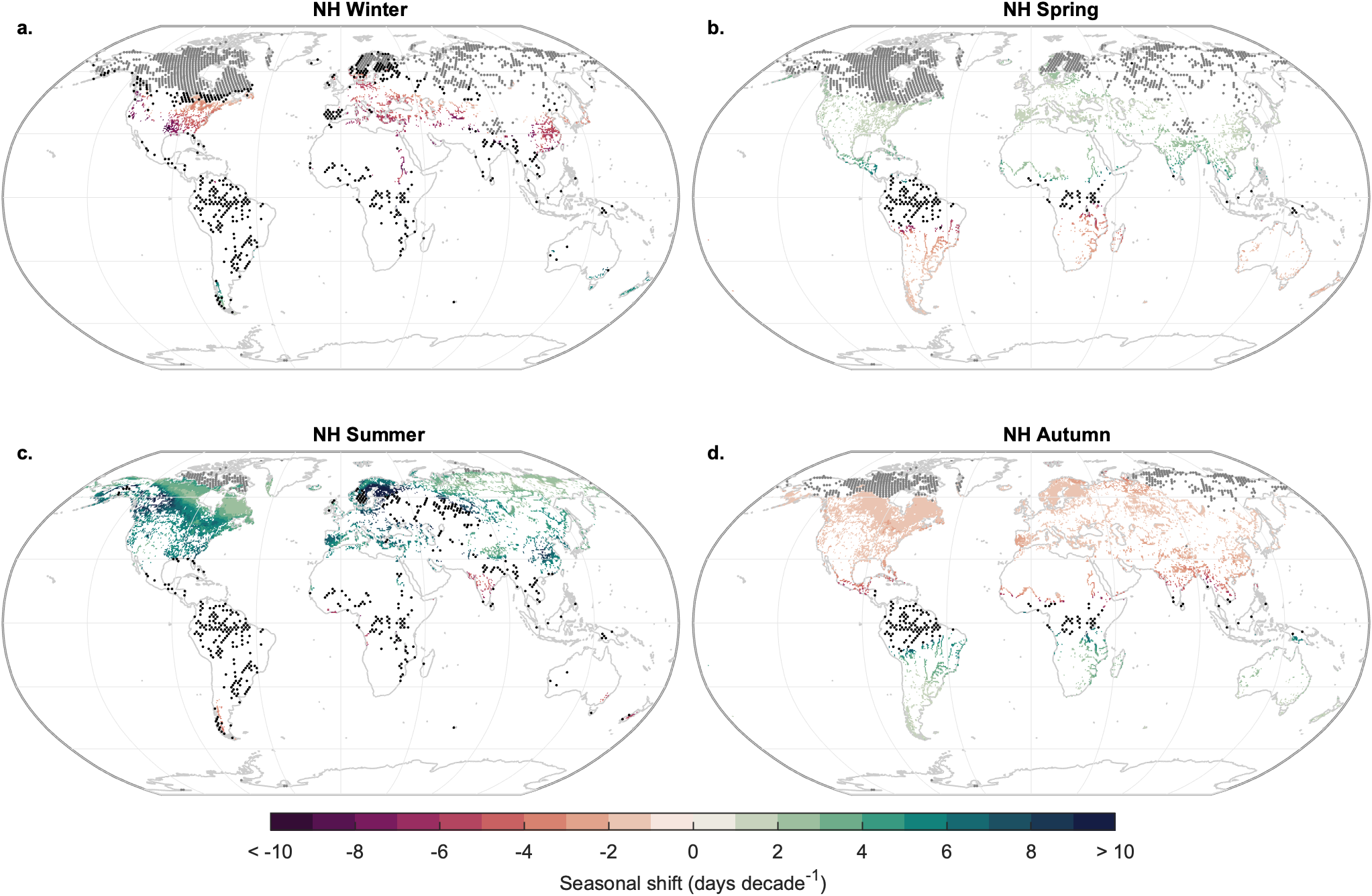
 **Figure S27 |** Shown are future (2021-2099) seasonal shifts (days decade^-1^) in the timing of **(a)** winter (December-February in the Northern Hemisphere [NH] and June-August in Southern Hemisphere [SH]), (**b**) spring (March-May in NH and September-November in the SH), **(c)** summer (June-August in the NH and December-February in SH), and **(d)** autumn (September-November in the NH and March-May in SH) in lakes worldwide under Representative Concentration Pathway (RCP) 6.0. Positive and negative values indicate when the timing of a season is projected to advance or be delayed, respectively. Stipple markings represents regions that were not included in the analysis, either due to the presence of lake ice cover or when lakes experienced a minimal (< 0.5 °C month^-1^) seasonal rate of change in surface water temperature during the season of interest.


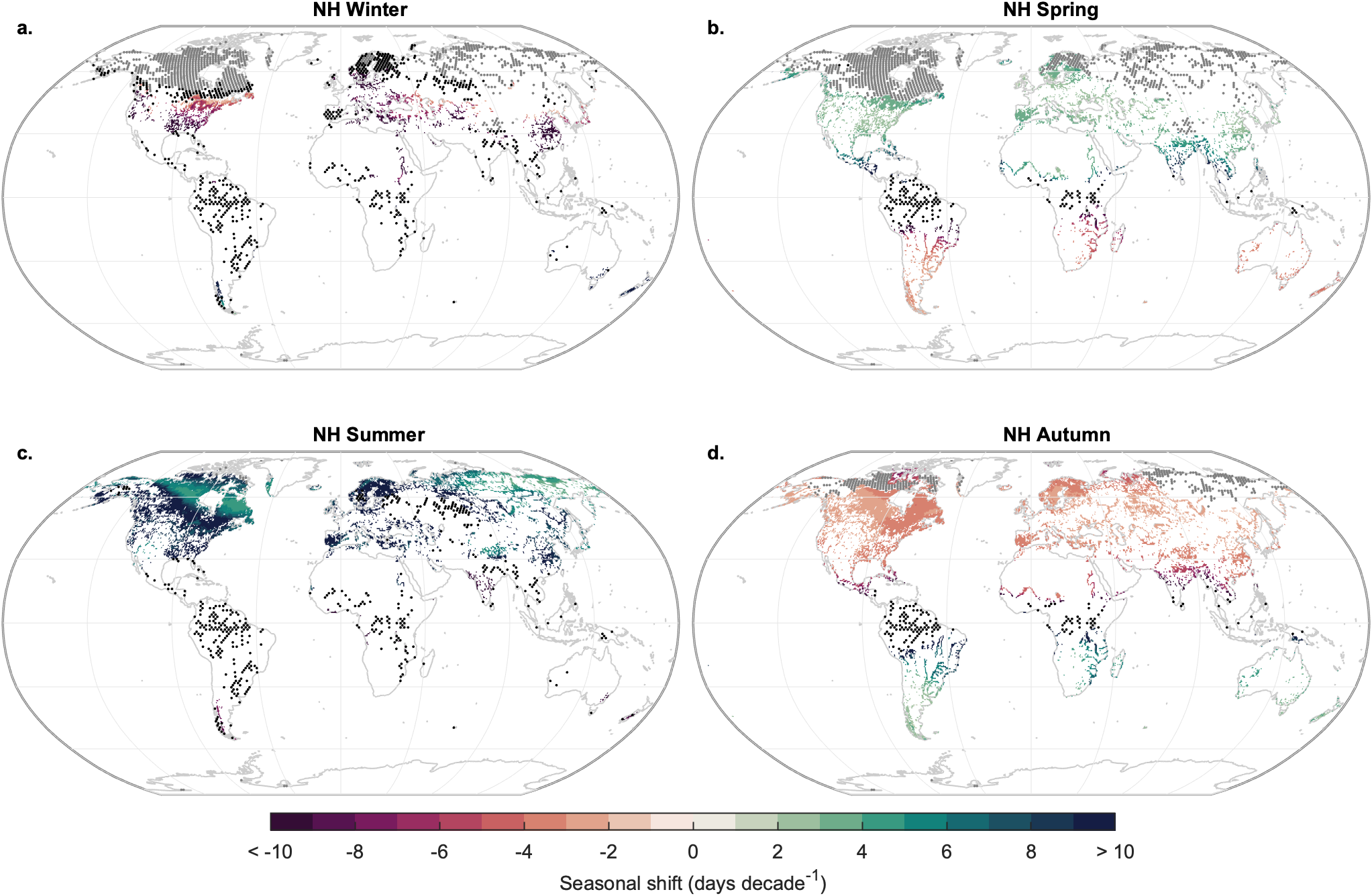
 **Figure S28 |** Shown are future (2021-2099) seasonal shifts (days decade^-1^) in the timing of **(a)** winter (December-February in the Northern Hemisphere [NH] and June-August in Southern Hemisphere [SH]), (**b**) spring (March-May in NH and September-November in the SH), **(c)** summer (June-August in the NH and December-February in SH), and **(d)** autumn (September-November in the NH and March-May in SH) in lakes worldwide under Representative Concentration Pathway (RCP) 8.5. Positive and negative values indicate when the timing of a season is projected to advance or be delayed, respectively. Stipple markings represents regions that were not included in the analysis, either due to the presence of lake ice cover or when lakes experienced a minimal (< 0.5 °C month^-1^) seasonal rate of change in surface water temperature during the season of interest.

**
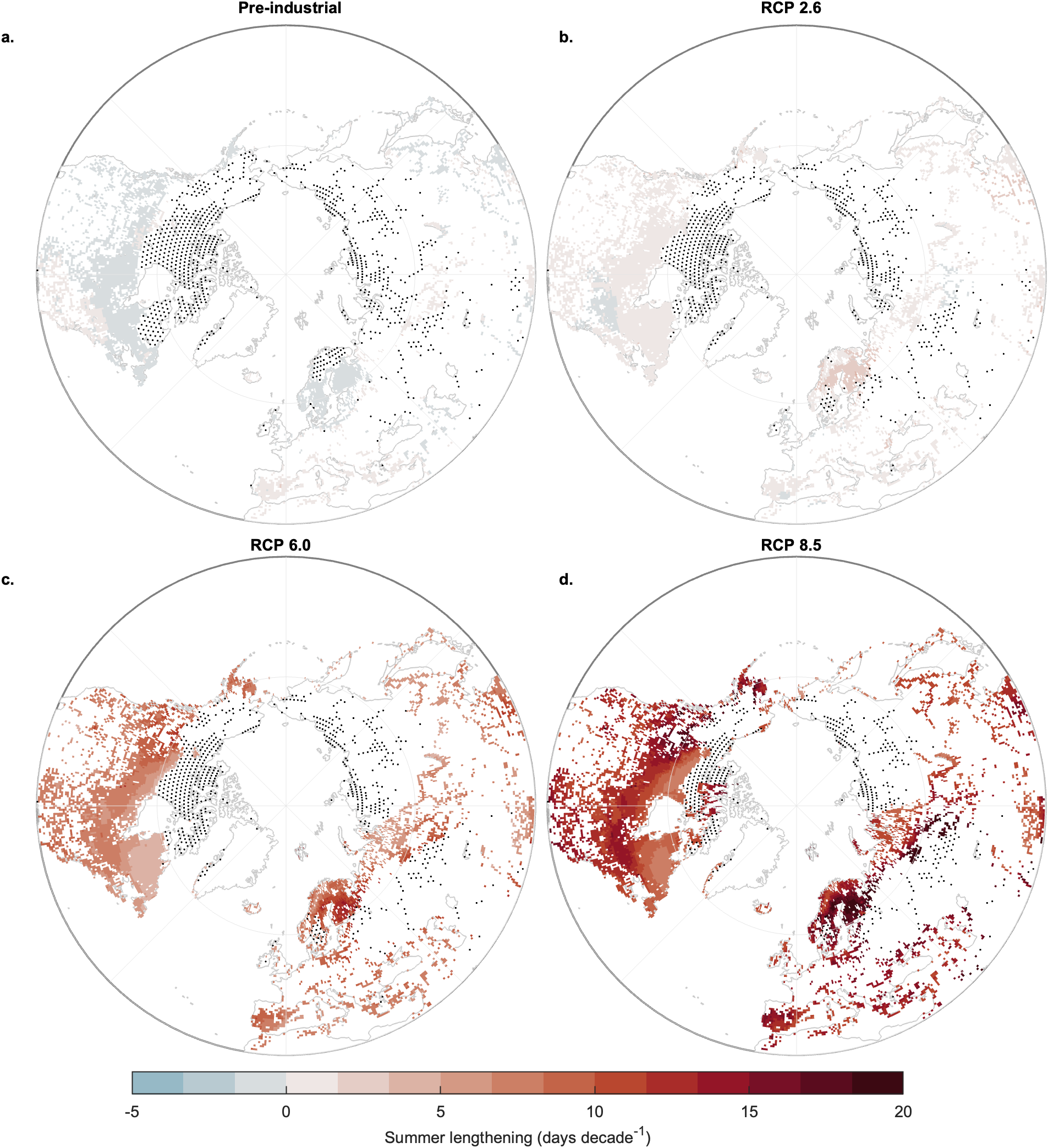
**

**Figure S29 |** Shown are future (2021-2099) changes to the duration of summer under (**a**) a pre-industrial climatic forcing, (**b**) Representative Concentration Pathway (RCP) 2.6, (**c**) RCP 6.0, and (**d**) RCP 8.5. Positive and negative values indicate when the summer season is projected to lengthen or shorten, respectively. Stipple markings represents regions that were not included in the analysis, either due to the presence of lake ice cover or when lakes experienced a minimal (< 0.5 °C month^-1^) seasonal rate of change in surface water temperature during the season of interest.

**Table S1 |** Seasonal shifts in the timing of spring and autumn in lakes. Shown are the median and interquartile ranges (25^th^ and 75^th^) in the calculated shifts (days decade^-1^) in the timing of spring (March-May in Northern Hemisphere and September-November in the Southern Hemisphere), and autumn (September-November in the Northern Hemisphere and March-May in Southern Hemisphere) in lakes worldwide during the historic to contemporary period (1980-2021). Positive and negative values indicate when the timing of a season has advanced or been delayed, respectively. Also shown are the calculated seasonal shifts in the timing of spring and autumn in local air temperature (i.e., the longitude-latitude grid in which each representative lake is located).

|  | **Spring** | | | **Autumn** | | |
| --- | --- | --- | --- | --- | --- | --- |
|  | **Median** | **25^th^** | **75^th^** | **Median** | **25^th^** | **75^th^** |
| **Northern Hemisphere lakes** | 2.0 | 1.4 | 2.8 | -1.5 | -2.5 | -0.8 |
| **Southern Hemisphere lakes** | 1.8 | 0.7 | 3.4 | -1.1 | -1.9 | -0.5 |
| **Northern Hemisphere air** | 2.5 | 1.3 | 4.0 | -1.8 | -2.9 | -1.0 |
| **Southern Hemisphere air** | 3.6 | 1.0 | 8.1 | -1.6 | -3.2 | -0.5 |

**Table S2 |** Seasonal shifts in the timing of summer in lakes. Shown are the median and interquartile ranges (25^th^ and 75^th^) in the calculated shifts (days decade^-1^) in the timing of summer (June-August in Northern Hemisphere and December-February in the Southern Hemisphere) in lakes worldwide during the historic to contemporary period (1980-2021). Positive and negative values indicate when the timing of a season has advanced or been delayed, respectively. Also shown are the calculated seasonal shifts in the timing of summer in local air temperature (i.e., the longitude-latitude grid in which each representative lake is located).

|  | **Median** | **25^th^** | **75^th^** |
| --- | --- | --- | --- |
| **Northern Hemisphere lakes** | 4.6 | 2.4 | 7.0 |
| **Southern Hemisphere lakes** | 6.4 | 3.1 | 10.0 |
| **Northern Hemisphere air** | 9.0 | -0.7 | 17.5 |
| **Southern Hemisphere air** | 13.1 | 3.8 | 24.2 |

**Table S3 |** Alterations to the duration of summer in lakes. Shown are the median and interquartile ranges (25^th^ and 75^th^) in the calculated changes to the duration of summer, estimated as the difference between the seasonal shifts in the start of summer (June-August in Northern Hemisphere and December-February in the Southern Hemisphere) and the start of autumn (September-November in the Northern Hemisphere and March-May in Southern Hemisphere) in lakes during the historic to contemporary period (1980-2021). Positive and negative values indicate when the summer season has lengthened or shortened, respectively. Also shown are the calculated alterations in the duration of summer in local air temperature (i.e., the longitude-latitude grid in which each representative lake is located).

|  | **Median** | **25^th^** | **75^th^** |
| --- | --- | --- | --- |
| **Northern Hemisphere lakes** | 5.6 | 3.6 | 8.9 |
| **Southern Hemisphere lakes** | 7.6 | 4.3 | 10.8 |
| **Northern Hemisphere air** | 11.1 | 5.3 | 18.4 |
| **Southern Hemisphere air** | 13.6 | 4.9 | 26.0 |

**Table S4 |** The pace of shifting seasons in the North American Great Lakes. Shown are the median and interquartile ranges (25^th^ and 75^th^) in the calculated shifts (days decade^-1^) in the timing of summer (June-August) and autumn (September-November) during the satellite data-taking period (1995-2021). Positive and negative values indicate when the timing of a season has advanced or been delayed, respectively. Also shown are calculated changes to the duration of summer, which was estimated as the difference between seasonal shifts in the start of summer and autumn. Positive and negative values indicate when the summer season has lengthened or shortened, respectively.

|  | **Summer** | | | **Autumn** | | | **Summer lengthening** | | |
| --- | --- | --- | --- | --- | --- | --- | --- | --- | --- |
|  | **Median** | **25^th^** | **75^th^** | **Median** | **25^th^** | **75^th^** | **Median** | **25^th^** | **75^th^** |
| **Superior** | 3.9 | 2.5 | 5.1 | -5.1 | -6.0 | -3.9 | 8.5 | 7.4 | 9.6 |
| **Michigan** | 4.2 | 3.1 | 5.3 | -3.5 | -5.1 | -2.5 | 8.1 | 5.6 | 9.8 |
| **Huron** | 4.9 | 3.9 | 6.5 | -4.0 | -5.4 | -2.8 | 8.6 | 7.4 | 10.7 |
| **Erie** | 5.5 | 4.8 | 6.5 | -3.5 | -3.9 | -3.1 | 8.9 | 8.3 | 9.9 |
| **Ontario** | 6.8 | 5.9 | 8.5 | -4.2 | -5.0 | -3.6 | 11.0 | 9.6 | 13.2 |

**Table S5 |** Shown are the median shifts (days decade^-1^) in the timing of the seasons during the satellite data-taking period (1995-2021) within the Great Lakes defined according to three-month moving windows from March to November. Positive and negative values indicate when the timing of a season has advanced or been delayed, respectively. Also shown in brackets is the percentage of lake pixels that were used for calculating the seasonal shifts. Within-lake pixels that were excluded from the analysis include those that were either ice covered or had a minimal (< 0.5 °C month^-1^) seasonal rate of change in surface water temperature during the season of interest.

|  | **Mar-May** | **Apr-Jun** | **May-Jul** | **Jun-Aug** | **Jul-Sep** | **Aug-Oct** | **Sep-Nov** |
| --- | --- | --- | --- | --- | --- | --- | --- |
| **Superior** | - (0) | - (0) | 3.8 (52) | 3.4 (100) | 5.8 (84) | -0.4 (100) | -5.1 (93) |
| **Michigan** | 8.34(10) | 3.8 (55) | 1.5 (99) | 4.2 (100) | -6.5 (26) | -2.2 (100) | -3.5 (95) |
| **Huron** | - (0) | - (0) | 1.4 (65) | 4.9 (100) | -5.4 (38) | -2.3 (100) | -4.0 (88) |
| **Erie** | - (0) | - (0) | 1.3 (99) | 5.5 (100) | -9.7 (78) | -4.1 (100) | -3.5 (99) |
| **Ontario** | 10.1 (9) | 4.0 (72) | 1.9 (100) | 6.8 (100) | -16.5 (30) | -4.8 (100) | -4.2 (99) |

**Table S6 |** Shown are the median shifts (days decade^-1^) in the timing of the seasons in lakes worldwide during the historic to contemporary period (1980-2021), defined according to three-month moving windows. Positive and negative values indicate when the timing of a season has advanced or been delayed, respectively. Also shown in brackets is the percentage of lakes that were used for calculating the seasonal shifts. Lakes excluded from the analysis include those that were either ice covered or had a minimal (< 0.5 °C month^-1^) seasonal rate of change in surface water temperature during the season of interest.

| **Season** | **Northern Hemisphere lakes** | **Southern Hemisphere lakes** |
| --- | --- | --- |
| **Jan-Mar** | 3.7 (19) | -2.9 (38) |
| **Feb-Apr** | 2.8 (24) | -1.4 (62) |
| **Mar-May** | 2.0 (26) | -1.1 (75) |
| **Apr-Jun** | 1.7 (31) | 0.2 (85) |
| **May-Jul** | 1.7 (43) | -1.4 (88) |
| **Jun-Aug** | 4.4 (50) | -2.6 (37) |
| **Jul-Sep** | -2.4 (75) | 1.1 (76) |
| **Aug-Oct** | -1.8 (53) | 1.3 (88) |
| **Sep-Nov** | -1.5 (34) | 1.9 (80) |
| **Oct-Dec** | -1.7 (25) | 2.0 (69) |
| **Nov-Jan** | -2.4 (23) | 1.9 (58) |
| **Dec-Feb** | -3.5 (15) | 6.4 (27) |

**Table S7 |** Future shifts in the timing of spring and autumn in lakes. Shown are the median and interquartile ranges (25^th^ and 75^th^) in the calculated future (2021-2099) shifts (days decade^-1^) in the timing of spring (March-May in Northern Hemisphere [NH] and September-November in the Southern Hemisphere [SH]), and autumn (September-November in the NH and March-May in SH) under different Representative Concentration Pathway (RCP) scenarios (2.6, 6.0 and 8.5) as well as the pre-industrial (PI) control simulation. Positive and negative values indicate when the timing of a season has advanced or been delayed, respectively.

|  |  | **Spring** | | | **Autumn** | | |
| --- | --- | --- | --- | --- | --- | --- | --- |
|  |  | **Median** | **25^th^** | **75^th^** | **Median** | **25^th^** | **75^th^** |
| **PI** | **Northern Hemisphere lakes** | -0.0 | -0.2 | 0.2 | 0.0 | -0.1 | 0.1 |
|  | **Southern Hemisphere lakes** | -0.2 | -0.3 | -0.1 | 0.2 | 0.0 | 0.3 |
| **RCP 2.6** | **Northern Hemisphere lakes** | 0.3 | 0.1 | 0.5 | -0.2 | -0.3 | -0.1 |
|  | **Southern Hemisphere lakes** | -0.2 | -0.4 | -0.1 | 0.2 | 0.1 | 0.4 |
| **RCP 6.0** | **Northern Hemisphere lakes** | 1.8 | 1.6 | 2.4 | -1.7 | -1.9 | -1.5 |
|  | **Southern Hemisphere lakes** | -2.1 | -3.1 | -1.6 | 2.5 | 1.6 | 3.9 |
| **RCP 8.5** | **Northern Hemisphere lakes** | 3.3 | 2.8 | 4.0 | -3.1 | -3.4 | -2.6 |
|  | **Southern Hemisphere lakes** | -3.8 | -5.6 | -3.0 | 4.7 | 3.0 | 7.1 |

**Table S8 |** Future shifts in the timing of summer in lakes. Shown are the median and interquartile ranges (25^th^ and 75^th^) in the calculated future (2021-2099) shifts (days decade^-1^) in the timing of summer (June-August in Northern Hemisphere [NH] and December-February in the Southern Hemisphere [SH]) under different Representative Concentration Pathway (RCP) scenarios (2.6, 6.0 and 8.5) as well as the pre-industrial (PI) control simulation. Positive and negative values indicate when the timing of a season has advanced or been delayed, respectively.

|  |  | **Median** | **25^th^** | **75^th^** |
| --- | --- | --- | --- | --- |
| **PI** | **Northern Hemisphere lakes** | -0.0 | -0.3 | 0.2 |
|  | **Southern Hemisphere lakes** | -0.1 | -0.3 | 0.1 |
| **RCP 2.6** | **Northern Hemisphere lakes** | 0.5 | 0.3 | 0.8 |
|  | **Southern Hemisphere lakes** | -0.2 | -0.7 | -0.1 |
| **RCP 6.0** | **Northern Hemisphere lakes** | 4.8 | 3.2 | 6.1 |
|  | **Southern Hemisphere lakes** | -4.0 | -5.6 | -3.0 |
| **RCP 8.5** | **Northern Hemisphere lakes** | 8.3 | 5.9 | 10.2 |
|  | **Southern Hemisphere lakes** | -8.7 | -10.9 | -7.7 |

**Table S9 |** Future alterations to the duration of summer in lakes. Shown are the median and interquartile ranges (25^th^ and 75^th^) in the calculated future (2021-2099) changes to the duration of summer, estimated as the difference between the seasonal shifts in the start of summer (June-August in Northern Hemisphere [NH] and December-February in the Southern Hemisphere [SH]) and the start of autumn (September-November in the NH and March-May in SH) under different Representative Concentration Pathway (RCP) scenarios (2.6, 6.0 and 8.5) as well as the pre-industrial (PI) control simulation. Positive and negative values indicate when the summer season has lengthened or shortened, respectively.

|  |  | **Median** | **25^th^** | **75^th^** |
| --- | --- | --- | --- | --- |
| **PI** | **Northern Hemisphere lakes** | -0.2 | -0.5 | 0.1 |
|  | **Southern Hemisphere lakes** | 0.0 | -0.2 | 1.0 |
| **RCP 2.6** | **Northern Hemisphere lakes** | 0.8 | 0.4 | 1.1 |
|  | **Southern Hemisphere lakes** | -0.4 | -0.9 | 0.1 |
| **RCP 6.0** | **Northern Hemisphere lakes** | 7.2 | 6.0 | 8.3 |
|  | **Southern Hemisphere lakes** | 7.0 | 5.9 | 7.5 |
| **RCP 8.5** | **Northern Hemisphere lakes** | 12.1 | 10.3 | 13.8 |
|  | **Southern Hemisphere lakes** | 14.0 | 12.3 | 15.2 |

**Table S10 |** Seasonal shifts in the timing of spring in lakes. Shown are future (2021-2099) seasonal shifts (days decade^-1^) in the timing of spring (March-May in Northern Hemisphere and September-November in the Southern Hemisphere) in 50 globally distributed lakes under Representative Concentration Pathway (RCP) 2.6, 6.0 and 8.5, as well as a pre-Industrial control simulation. Positive and negative values indicate when the timing of a season is projected to advance or be delayed, respectively. Missing values (-) represents lakes that experienced either the presence of lake ice cover or when they experience a minimal (< 0.5 °C month^-1^) seasonal rate of change in surface water temperature during the season of interest.

| **Lake** | **Country** | **Pre-Industrial** | **RCP 2.6** | **RCP 6.0** | **RCP 8.5** |
| --- | --- | --- | --- | --- | --- |
| **Lake Tarawera** | New Zealand | 0.6 | -0.2 | 3.2 | 5.9 |
| **Lake Rotorua** | New Zealand | 0.4 | -0.2 | 1.8 | 3.6 |
| **Lake Burley Griffin** | Australia | 0.3 | 0.1 | 1.7 | 2.9 |
| **Mt Bold** | Australia | 0.3 | 0.1 | 1.8 | 3.6 |
| **Lake Kinneret** | Israel | -0.2 | 0.2 | 2.6 | 3.9 |
| **Falling Creek Reservoir** | USA | 0.3 | 0.1 | 1.3 | 2.4 |
| **Alqueva Reservoir** | USA | -0.2 | 0.2 | 1.6 | 3.3 |
| **Lake Tahoe** | USA | 0.0 | 0.3 | 5.9 | 9.8 |
| **Laramie Lake** | USA | 0.0 | -0.4 | 1.4 | 2.2 |
| **Sau Reservoir** | Spain | 0.0 | 0.4 | 1.8 | 3.8 |
| **Delavan Lake** | USA | 0.2 | -0.1 | 1.1 | 2.4 |
| **Lake Wingra** | USA | 0.0 | -0.0 | 1.3 | 2.2 |
| **Lake Monona** | USA | -0.1 | -0.1 | 0.8 | 2.1 |
| **Lake Mendota** | USA | - | - | - | - |
| **Okauchee Lake** | USA | -0.2 | 0.1 | 1.4 | 2.7 |
| **Lake Sunapee** | USA | - | - | 1.5 | 3.3 |
| **Fish Lake** | USA | 0.0 | 0.0 | 1.0 | 2.3 |
| **Green Lake** | USA | 0.0 | -0.1 | 1.7 | 3.1 |
| **Great Pond** | USA | -0.4 | -0.1 | 1.3 | 2.4 |
| **Eagle Lake** | Canada | - | - | - | - |
| **Harp Lake** | Canada | - | - | - | 2.8 |
| **Lake Bourget** | France | 0.2 | 0.1 | 2.1 | 4.0 |
| **Two Sisters Lake** | USA | - | - | - | - |
| **Lake Annecy** | France | 0.2 | 0.3 | 1.6 | 3.4 |
| **Crystal Lake** | USA | - | - | - | - |
| **Crystal Bog** | USA | -0.1 | 0.1 | 0.6 | 1.2 |
| **Sparkling Lake** | USA | - | - | - | - |
| **Trout Lake** | USA | - | - | - | - |
| **Allequash Lake** | USA | -0.2 | -0.1 | 1.4 | 2.2 |
| **Black Oak Lake** | USA | - | - | - | - |
| **Lake Geneva** | France/Switzerland | 0.2 | 0.4 | 2.5 | 4.2 |
| **Lake Neuchatel** | Switzerland | 0.1 | 0.3 | 1.9 | 3.8 |
| **Lake Biel** | Switzerland | 0.3 | 0.3 | 1.7 | 3.2 |
| **Lower Lake Zurich** | Switzerland | 0.2 | 0.4 | 2.3 | 4.1 |
| **Lake Sammamish** | USA | -0.4 | 0.7 | 2.5 | 4.1 |
| **Lake Washington** | USA | -0.3 | 0.9 | 3.5 | 5.8 |
| **Rimov Reservoir** | Czechia | - | 0.4 | 1.2 | 2.4 |
| **Zlutice Reservoir** | Czechia | - | - | - | - |
| **Rappbode Reservoir** | Germany | -0.2 | 0.2 | 1.5 | 2.5 |
| **Lake Mueggelsee** | Germany | -0.1 | 0.3 | 1.1 | 1.7 |
| **Lake Stechlin** | Germany | -0.4 | 0.1 | 1.6 | 2.0 |
| **Lough Feeagh** | Ireland | -0.1 | 0.2 | 0.8 | 2.0 |
| **Windermere** | UK | 0.0 | 0.1 | 1.2 | 2.2 |
| **Esthwaite Water** | UK | 0.1 | 0.3 | 0.8 | 1.6 |
| **Lake Nohipalo Valgejrv** | Estonia | - | - | - | - |
| **Ekoln basin of Malaren** | Sweden | 1.2 | 0.0 | 2.7 | 3.6 |
| **Lake Erken** | Sweden | - | - | 2.0 | - |
| **Lake Kuivajarvi** | Finland | - | - | - | - |
| **Lake Paaijarvi** | Finland | - | - | - | - |
| **Lake Vendyurskoe** | Russia | -0.2 | -0.1 | 2.0 | 4.2 |

**Table S11 |** Seasonal shifts in the timing of summer in lakes. Shown are future (2021-2099) seasonal shifts (days decade^-1^) in the timing of summer (June-August in Northern Hemisphere and December-February in the Southern Hemisphere) in 50 globally distributed lakes under Representative Concentration Pathway (RCP) 2.6, 6.0 and 8.5, as well as a pre-Industrial control simulation. Positive and negative values indicate when the timing of a season is projected to advance or be delayed, respectively. Missing values (-) represents lakes that experienced either the presence of lake ice cover or when they experience a minimal (< 0.5 °C month^-1^) seasonal rate of change in surface water temperature during the season of interest.

| **Lake** | **Country** | **Pre-Industrial** | **RCP 2.6** | **RCP 6.0** | **RCP 8.5** |
| --- | --- | --- | --- | --- | --- |
| **Lake Tarawera** | New Zealand | 0.7 | 0.1 | 4.7 | 10.4 |
| **Lake Rotorua** | New Zealand | 0.7 | 0.6 | 5.5 | 12.5 |
| **Lake Burley Griffin** | Australia | 0.6 | -0.2 | 10.6 | 15.7 |
| **Mt Bold** | Australia | 0.6 | -1.8 | 6.1 | 13.4 |
| **Lake Kinneret** | Israel | -0.1 | 0.6 | 5.6 | 9.2 |
| **Falling Creek Reservoir** | USA | 0.7 | -0.5 | 8.7 | 13.2 |
| **Alqueva Reservoir** | USA | 0.2 | 0.4 | 5.2 | 8.1 |
| **Lake Tahoe** | USA | -0.2 | 0.3 | 3.5 | 5.9 |
| **Laramie Lake** | USA | -1.1 | 1.4 | 12.0 | 18.1 |
| **Sau Reservoir** | Spain | 0.9 | 0.5 | 4.3 | 7.1 |
| **Delavan Lake** | USA | -0.2 | -0.3 | 5.6 | 8.1 |
| **Lake Wingra** | USA | 0.1 | -1.1 | 10.0 | 14.6 |
| **Lake Monona** | USA | -0.1 | -0.3 | 6.3 | 9.1 |
| **Lake Mendota** | USA | -0.1 | -0.3 | 5.4 | 8.1 |
| **Okauchee Lake** | USA | -0.1 | -0.3 | 5.7 | 8.3 |
| **Lake Sunapee** | USA | 0.2 | 0.4 | 4.3 | 7.6 |
| **Fish Lake** | USA | -0.1 | -0.5 | 7.8 | 10.9 |
| **Green Lake** | USA | -0.1 | -0.1 | 4.6 | 7.2 |
| **Great Pond** | USA | 0.3 | 0.6 | 4.6 | 8.3 |
| **Eagle Lake** | Canada | 0.3 | -0.5 | 4.8 | 9.1 |
| **Harp Lake** | Canada | 0.6 | -0.7 | 6.1 | 11.2 |
| **Lake Bourget** | France | 0.4 | 0.7 | 5.0 | 10.3 |
| **Two Sisters Lake** | USA | 0.0 | 0.0 | 5.0 | 7.7 |
| **Lake Annecy** | France | 0.4 | 0.4 | 4.8 | 8.7 |
| **Crystal Lake** | USA | 0.0 | 0.0 | 5.1 | 8.1 |
| **Crystal Bog** | USA | 0.4 | -0.2 | 12.8 | 15.5 |
| **Sparkling Lake** | USA | 0.1 | 0.1 | 5.3 | 8.3 |
| **Trout Lake** | USA | 0.1 | 0.1 | 4.7 | 7.5 |
| **Allequash Lake** | USA | 0.2 | 0.0 | 7.5 | 11.2 |
| **Black Oak Lake** | USA | 0.2 | 0.0 | 5.1 | 8.1 |
| **Lake Geneva** | France/Switzerland | 0.3 | 0.5 | 4.5 | 6.8 |
| **Lake Neuchatel** | Switzerland | 0.2 | 0.1 | 4.3 | 7.2 |
| **Lake Biel** | Switzerland | 0.6 | 0.4 | 6.0 | 9.4 |
| **Lower Lake Zurich** | Switzerland | 0.6 | 0.3 | 5.0 | 7.8 |
| **Lake Sammamish** | USA | -0.6 | 1.0 | 5.7 | 9.1 |
| **Lake Washington** | USA | -0.5 | 1.0 | 5.1 | 8.4 |
| **Rimov Reservoir** | Czechia | 1.1 | 0.9 | 7.0 | 10.7 |
| **Zlutice Reservoir** | Czechia | 0.2 | 1.0 | 7.3 | 10.5 |
| **Rappbode Reservoir** | Germany | 0.6 | -0.2 | 6.2 | 9.9 |
| **Lake Mueggelsee** | Germany | 1.4 | 0.3 | 10.4 | 16.1 |
| **Lake Stechlin** | Germany | 0.2 | 0.1 | 6.9 | 10.2 |
| **Lough Feeagh** | Ireland | -1.5 | -0.3 | 3.0 | 9.1 |
| **Windermere** | UK | -1.8 | -0.8 | 6.2 | 12.5 |
| **Esthwaite Water** | UK | -3.0 | -1.7 | 9.3 | 18.2 |
| **Lake Nohipalo Valgejrv** | Estonia | 0.0 | 1.3 | 11.8 | 14.0 |
| **Ekoln basin of Malaren** | Sweden | 0.0 | 1.5 | 9.9 | 16.9 |
| **Lake Erken** | Sweden | 0.0 | 1.1 | 6.9 | 10.6 |
| **Lake Kuivajarvi** | Finland | -0.6 | 0.1 | 13.6 | 18.5 |
| **Lake Paaijarvi** | Finland | 1.1 | 0.7 | 10.0 | 14.5 |
| **Lake Vendyurskoe** | Russia | 0.2 | 3.1 | 16.7 | 20.2 |

**Table S12 |** Seasonal shifts in the timing of autumn in lakes. Shown are future (2021-2099) seasonal shifts (days decade^-1^) in the timing of autumn (September-November in the Northern Hemisphere and March-May in Southern Hemisphere) in 50 globally distributed lakes under Representative Concentration Pathway (RCP) 2.6, 6.0 and 8.5, as well as a pre-Industrial control simulation. Positive and negative values indicate when the timing of a season is projected to advance or be delayed, respectively. Missing values (-) represents lakes that experienced either the presence of lake ice cover or when they experience a minimal (< 0.5 °C month^-1^) seasonal rate of change in surface water temperature during the season of interest.

| **Lake** | **Country** | **Pre-Industrial** | **RCP 2.6** | **RCP 6.0** | **RCP 8.5** |
| --- | --- | --- | --- | --- | --- |
| **Lake Tarawera** | New Zealand | -0.1 | 0.6 | -2.9 | -5.5 |
| **Lake Rotorua** | New Zealand | -0.1 | 0.5 | -2.1 | -4.0 |
| **Lake Burley Griffin** | Australia | -0.2 | -0.1 | -1.5 | -2.9 |
| **Mt Bold** | Australia | -0.3 | -0.0 | -2.0 | -4.1 |
| **Lake Kinneret** | Israel | -0.1 | -0.5 | -2.3 | -4.1 |
| **Falling Creek Reservoir** | USA | 0.1 | -0.4 | -1.7 | -2.9 |
| **Alqueva Reservoir** | USA | 0.0 | -0.0 | -2.3 | -3.9 |
| **Lake Tahoe** | USA | 0.1 | 0.2 | -4.6 | -7.4 |
| **Laramie Lake** | USA | - | -0.6 | -1.3 | - |
| **Sau Reservoir** | Spain | -0.1 | -0.3 | -2.0 | -3.4 |
| **Delavan Lake** | USA | 0.0 | -0.3 | -1.7 | -2.7 |
| **Lake Wingra** | USA | 0.2 | -0.3 | -1.6 | -2.8 |
| **Lake Monona** | USA | 0.1 | -0.2 | -1.7 | -2.7 |
| **Lake Mendota** | USA | 0.1 | -0.2 | -1.7 | -2.8 |
| **Okauchee Lake** | USA | 0.2 | -0.2 | -1.7 | -2.8 |
| **Lake Sunapee** | USA | 0.1 | -0.6 | -2.1 | -3.4 |
| **Fish Lake** | USA | 0.1 | -0.2 | -1.7 | -2.6 |
| **Green Lake** | USA | 0.1 | -0.2 | -1.9 | -3.1 |
| **Great Pond** | USA | 0.1 | -0.2 | -1.8 | -3.1 |
| **Eagle Lake** | Canada | 0.2 | -0.3 | -1.6 | -3.2 |
| **Harp Lake** | Canada | 0.1 | -0.2 | -1.6 | -3.1 |
| **Lake Bourget** | France | 0.0 | -0.2 | -2.1 | -3.5 |
| **Two Sisters Lake** | USA | -0.1 | -0.3 | -1.5 | -3.2 |
| **Lake Annecy** | France | 0.1 | -0.2 | -2.0 | -3.4 |
| **Crystal Lake** | USA | 0.2 | -0.3 | -1.7 | -3.3 |
| **Crystal Bog** | USA | - | - | - | - |
| **Sparkling Lake** | USA | -0.1 | -0.3 | -1.6 | -2.9 |
| **Trout Lake** | USA | 0.1 | -0.3 | -1.7 | -3.0 |
| **Allequash Lake** | USA | - | - | - | - |
| **Black Oak Lake** | USA | 0.2 | -0.2 | -1.8 | -3.4 |
| **Lake Geneva** | France/Switzerland | 0.1 | -0.2 | -2.3 | -3.4 |
| **Lake Neuchatel** | Switzerland | 0.1 | -0.2 | -2.0 | -3.5 |
| **Lake Biel** | Switzerland | 0.1 | -0.2 | -1.8 | -3.2 |
| **Lower Lake Zurich** | Switzerland | 0.1 | -0.2 | -1.8 | -3.1 |
| **Lake Sammamish** | USA | 0.1 | -0.1 | -2.0 | -3.1 |
| **Lake Washington** | USA | 0.1 | -0.1 | -2.4 | -3.6 |
| **Rimov Reservoir** | Czechia | 0.0 | -0.2 | -1.4 | -2.8 |
| **Zlutice Reservoir** | Czechia | -0.3 | -0.1 | -1.3 | -2.8 |
| **Rappbode Reservoir** | Germany | 0.1 | -0.1 | -1.6 | -3.1 |
| **Lake Mueggelsee** | Germany | 0.2 | -0.3 | -1.1 | -2.6 |
| **Lake Stechlin** | Germany | 0.1 | -0.2 | -1.4 | -2.9 |
| **Lough Feeagh** | Ireland | 0.1 | 0.0 | -1.5 | -2.9 |
| **Windermere** | UK | 0.1 | 0.0 | -1.7 | -2.8 |
| **Esthwaite Water** | UK | 0.0 | 0.0 | -1.5 | -2.6 |
| **Lake Nohipalo Valgejrv** | Estonia | - | - | - | -3.0 |
| **Ekoln basin of Malaren** | Sweden | -0.2 | -0.3 | -1.2 | -2.6 |
| **Lake Erken** | Sweden | - | -0.4 | -1.2 | -2.6 |
| **Lake Kuivajarvi** | Finland | - | - | - | - |
| **Lake Paaijarvi** | Finland | - | - | -0.6 | -3.1 |
| **Lake Vendyurskoe** | Russia | - | - | - | - |

**Table S13 |** Future alterations to the duration of summer in lakes. Shown are future (2021-2099) changes (days decade^-1^) to the duration of summer, which was estimated as the difference between the seasonal shift in the start of summer and the start of autumn, in 50 globally distributed lakes under Representative Concentration Pathway (RCP) 2.6, 6.0 and 8.5, as well as a pre-Industrial control simulation. Positive and negative values indicate when the summer season is projected to lengthen or shorten, respectively. Missing values (-) represents lakes that experienced either the presence of lake ice cover or when they experience a minimal (< 0.5 °C month^-1^) seasonal rate of change in surface water temperature during the season of interest.

| **Lake** | **Country** | **Pre-Industrial** | **RCP 2.6** | **RCP 6.0** | **RCP 8.5** |
| --- | --- | --- | --- | --- | --- |
| **Lake Tarawera** | New Zealand | 0.8 | -0.4 | 7.6 | 15.9 |
| **Lake Rotorua** | New Zealand | 0.7 | 0.1 | 7.6 | 16.5 |
| **Lake Burley Griffin** | Australia | 0.8 | -0.1 | 12.1 | 18.5 |
| **Mt Bold** | Australia | 0.8 | -1.8 | 8.2 | 17.5 |
| **Lake Kinneret** | Israel | 0.0 | 1.1 | 8.0 | 13.4 |
| **Falling Creek Reservoir** | USA | 0.6 | -0.1 | 10.4 | 16.1 |
| **Alqueva Reservoir** | USA | 0.2 | 0.5 | 7.5 | 12.0 |
| **Lake Tahoe** | USA | -0.3 | 0.1 | 8.1 | 13.3 |
| **Laramie Lake** | USA | - | 1.6 | 13.4 | - |
| **Sau Reservoir** | Spain | 1.0 | 0.8 | 6.3 | 10.5 |
| **Delavan Lake** | USA | -0.2 | 0.0 | 7.3 | 10.8 |
| **Lake Wingra** | USA | -0.1 | -0.9 | 11.7 | 17.4 |
| **Lake Monona** | USA | -0.1 | -0.1 | 7.9 | 11.8 |
| **Lake Mendota** | USA | -0.3 | -0.0 | 7.1 | 10.9 |
| **Okauchee Lake** | USA | -0.2 | -0.1 | 7.4 | 11.1 |
| **Lake Sunapee** | USA | 0.1 | 1.0 | 6.4 | 11.0 |
| **Fish Lake** | USA | -0.2 | -0.2 | 9.5 | 13.6 |
| **Green Lake** | USA | -0.3 | 0.1 | 6.5 | 10.2 |
| **Great Pond** | USA | 0.2 | 0.8 | 6.4 | 11.3 |
| **Eagle Lake** | Canada | 0.1 | -0.2 | 6.4 | 12.3 |
| **Harp Lake** | Canada | 0.5 | -0.5 | 7.7 | 14.3 |
| **Lake Bourget** | France | 0.4 | 0.9 | 7.1 | 13.8 |
| **Two Sisters Lake** | USA | 0.1 | 0.3 | 6.5 | 10.9 |
| **Lake Annecy** | France | 0.4 | 0.6 | 6.8 | 12.0 |
| **Crystal Lake** | USA | -0.1 | 0.3 | 6.8 | 11.4 |
| **Crystal Bog** | USA | - | - | - | - |
| **Sparkling Lake** | USA | 0.1 | 0.4 | 6.8 | 11.2 |
| **Trout Lake** | USA | -0.1 | 0.3 | 6.4 | 10.5 |
| **Allequash Lake** | USA | - | - | - | - |
| **Black Oak Lake** | USA | 0.0 | 0.3 | 6.9 | 11.5 |
| **Lake Geneva** | France/Switzerland | 0.2 | 0.7 | 6.7 | 10.2 |
| **Lake Neuchatel** | Switzerland | 0.1 | 0.3 | 6.3 | 10.7 |
| **Lake Biel** | Switzerland | 0.5 | 0.6 | 7.8 | 12.5 |
| **Lower Lake Zurich** | Switzerland | 0.5 | 0.5 | 6.8 | 11.0 |
| **Lake Sammamish** | USA | -0.6 | 1.1 | 7.7 | 12.2 |
| **Lake Washington** | USA | -0.6 | 1.1 | 7.4 | 12.0 |
| **Rimov Reservoir** | Czechia | 1.1 | 1.2 | 8.5 | 13.5 |
| **Zlutice Reservoir** | Czechia | 0.6 | 1.1 | 8.7 | 13.3 |
| **Rappbode Reservoir** | Germany | 0.6 | -0.1 | 7.8 | 13.0 |
| **Lake Mueggelsee** | Germany | 1.2 | 0.6 | 11.5 | 18.7 |
| **Lake Stechlin** | Germany | 0.1 | 0.3 | 8.4 | 13.1 |
| **Lough Feeagh** | Ireland | -1.6 | -0.3 | 4.5 | 12.0 |
| **Windermere** | UK | -1.9 | -0.8 | 7.9 | 15.4 |
| **Esthwaite Water** | UK | -3.0 | -1.7 | 10.9 | 20.7 |
| **Lake Nohipalo Valgejrv** | Estonia | - | - | - | 17.1 |
| **Ekoln basin of Malaren** | Sweden | 0.2 | 1.8 | 11.1 | 19.4 |
| **Lake Erken** | Sweden | - | 1.4 | 8.1 | 13.2 |
| **Lake Kuivajarvi** | Finland | - | - | - | - |
| **Lake Paaijarvi** | Finland | - | - | 10.6 | 17.5 |
| **Lake Vendyurskoe** | Russia | - | - | - | - |
